# Update of gene expression/methylation and MiRNA profiling in colorectal cancer; application in diagnosis, prognosis, and targeted therapy

**DOI:** 10.1371/journal.pone.0265527

**Published:** 2022-03-25

**Authors:** Amir Mehrgou, Shahram Teimourian

**Affiliations:** 1 Department of Medical Genetics and Molecular Biology, School of Medicine, Iran University of Medical Sciences, Tehran, Iran; 2 Department of Medical Genetics, School of Medicine, Iran University of Medical Sciences, Tehran, Iran; Institute of Parasitology and Biomedicine, SPAIN

## Abstract

**Background:**

Colorectal cancer is one of the most deadliest malignancies worldwide. Due to the dearth of appropriate biomarkers, the diagnosis of this mortal disease is usually deferred, in its turn, culminating in the failure of prevention. By the same token, proper biomarkers are at play in determining the quality of prognosis. In other words, the survival rate is contingent upon the regulation of such biomarkers.

**Materials and methods:**

The information regarding expression (GSE41258, and GSE31905), methylation (GSE101764), and miRNA (dbDEMC) were downloaded. MEXPRESS and GEPIA confirmed the validated differentially expressed/methylated genes using TCGA data. Taking advantage of the correlation plots and receiver-operating-characteristic (ROC) curves, expression and methylation profiles were compared. The interactions between validated differentially expressed genes and differentially expressed miRNA were recognized and visualized by miRTarBase and Cytoscape, respectively. Then, the protein-protein interaction (PPI) network and hub genes were established via STRING and Cytohubba plugin. Utilizing R packages (DOSE, Enrichplot, and clusterProfiler) and DAVID database, the Functional Enrichment analysis and the detection of KEGG pathways were performed. Ultimately, in order to recognize the prognostic value of found biomarkers, they were evaluated through drawing survival plots for CRC patients.

**Results:**

In this research, we found an expression profile (with 13 novel genes), a methylation profile (with two novel genes), and a miRNA profile with diagnostic value. Concerning diagnosis, the expression profile was evaluated more powerful in comparison with the methylation profile. Furthermore, a prognosis-related expression profile was detected.

**Conclusion:**

In addition to diagnostic- and prognostic-applicability, the discerned profiles can assist in targeted therapy and current therapeutic strategies.

## 1. Introduction

Nowadays, colorectal cancer is one of the most rampant malignancies in the world [1–7]. Although the rate of mortality and morbidity of CRC is considered high, these rates have been decreased among people age more than 50 and on the other hand, this reduction has been compensated in less-than-50-year old people [2, 3]. Among the risk factors of CRC, we can point out population aging, the dearth of physical activity, obesity, abstinence of fruits and vegetables, alcohol consumption, tobacco, and the like [3]. The existence of heterogeneity leads to rough circumstances deferring the achievement of proper outcomes in CRC patients [2, 8, 9]. The quality of the prognosis of CRC patients is ultimately contingent on the time of diagnosis. The earlier the disease is diagnosed the more chance will be provided for patients to experience 5-year-survival [5, 10]. Since CRC development is a multiple-step phenomenon including, activation of oncogenes and inactivation of tumor suppressor genes, determining the molecular markers can play a main role as an excellent indicator for this disease [2, 5, 11, 12].

Aberrant methylation of DNA is one of the effective and culpable epigenetic phenomena in developing malignancies, such as CRC [13, 14]. Methylation can be observed in promoter, non-promoter, coding regions, and non-coding regions throughout the DNA sequence whereby, creates varied clinical features in CRC patients [13, 15]. Embryonic development, cell proliferation, and gene expression are under the control of methylation. Furthermore, methylation has the ability to dysregulate various cellular processes through silencing tumor suppressor genes culminating in malignancies, eventually [13]. Methylation is involved in controlling gene expression in both healthy and diseased statuses [16–18]. In most cases, methylation occurs in the vicinity of the transcription start site (TSS). However, even in promoters with a low density of CpG islands, methylation can regulate the corresponding gene expression [17, 19–21]. It is noteworthy that the methylation in non-promoter regions can modulate 1) splicing, 2) alternative gene transcript expression through an alternative promoter, and 3) activation of enhancers [22].

In the present study, we utilize microarray information so as to analyze methylation and expression profiles in colorectal cancer. Paving the way to reach our goal, we take advantage of R packages, MEXPRESS, GEPIA, correlation plot, ROC curve, Cytohubba plugin, and DAVID. Then, the possible interactions, which the found genes have with miRNAs, are investigated.

## 2. Results

### 2.1. Recognized Differentially Expressed Genes (DEGs), validated Differentially Expressed Genes (vDEGs), and Differentially Methylated Genes (DMGs)

**Fig 1:**
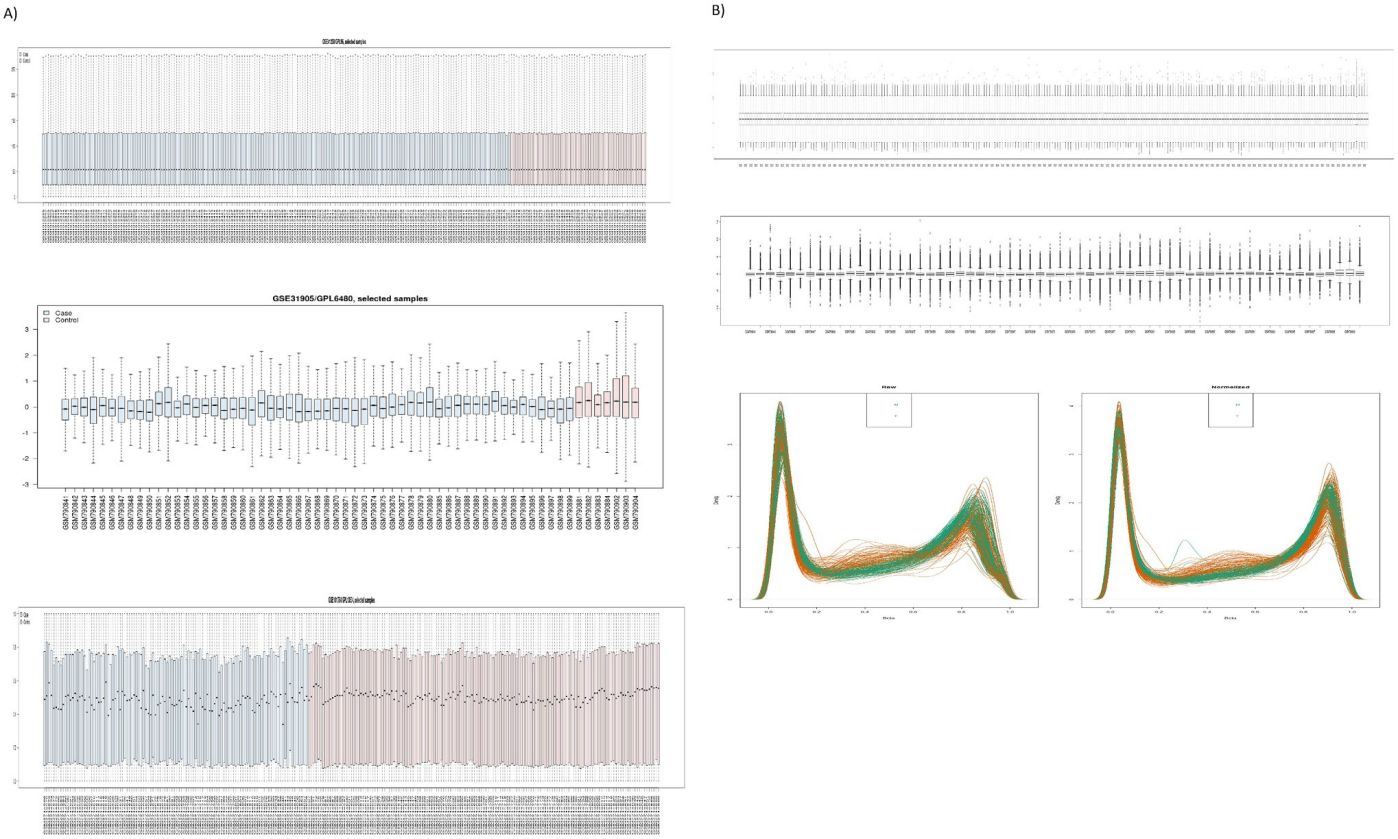
A) Box plots of pre-processed samples data in GEO2R. The top Box plots relate to the discovery dataset. The middle Box plots pertain to the validation dataset. The bottom Box plots are for the methylation dataset. B) Post-processed (Normalized) samples data. The top Box plots relate to the discovery dataset. The middle Box plots pertain to the validation dataset. The bottom left plot is for the methylation dataset raw data. The bottom right plot is for the methylation dataset normalized data.

**Fig 2:**
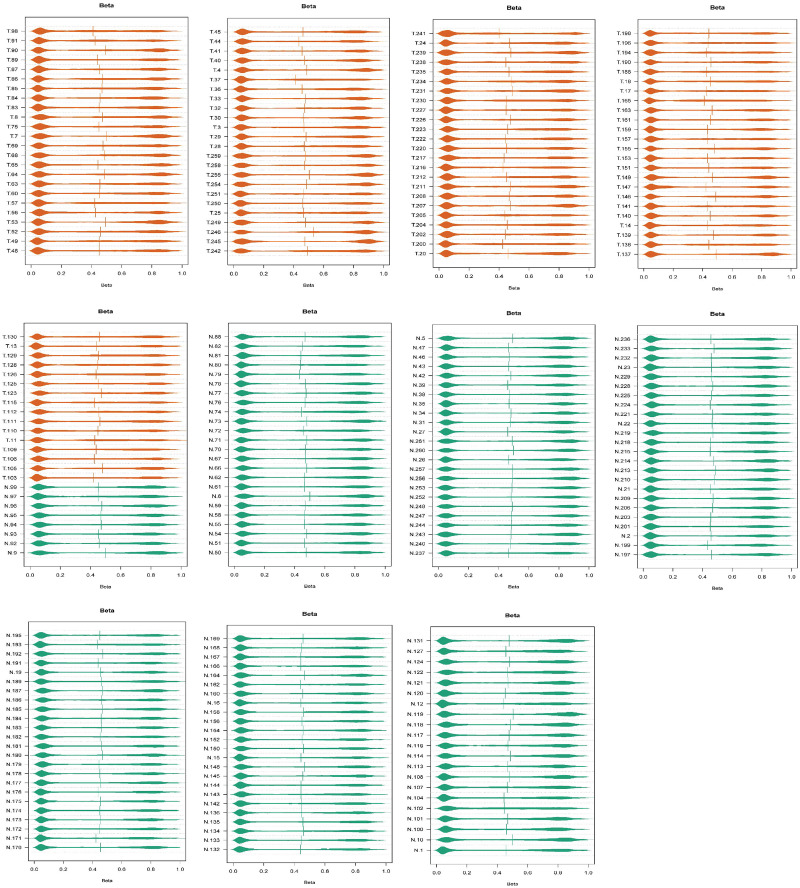
The quality control plots of normal and cancerous samples prior to analysis. As it is demonstrated the most of methylation alterations have occurred in two extrem sides (hypo-methylation and hyper-methylation).

**Fig 3:**
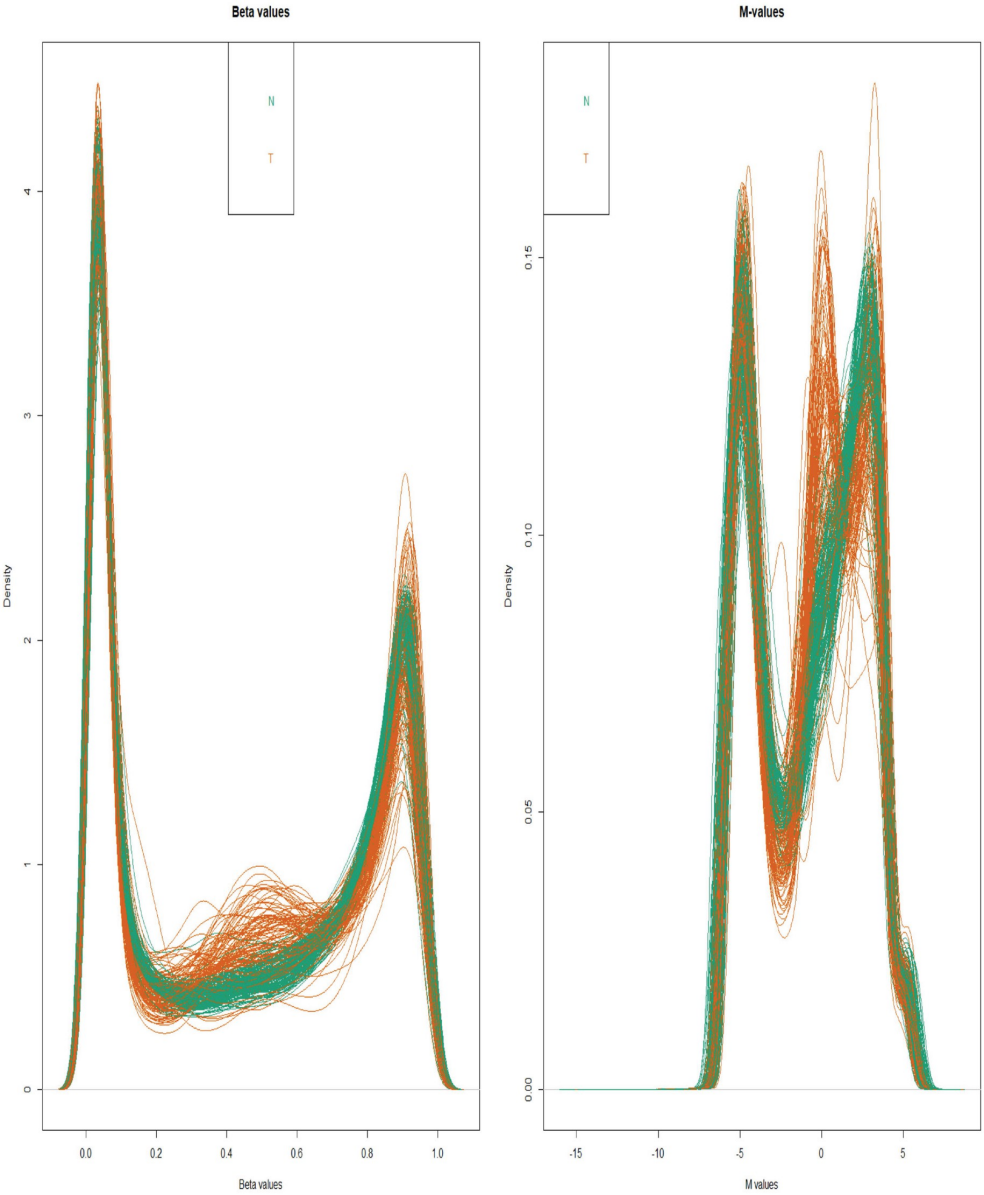
Comparison between Beta and M values. Beta and M values are usually used for visualization and statistical calculations, respectively.

**Fig 4:**
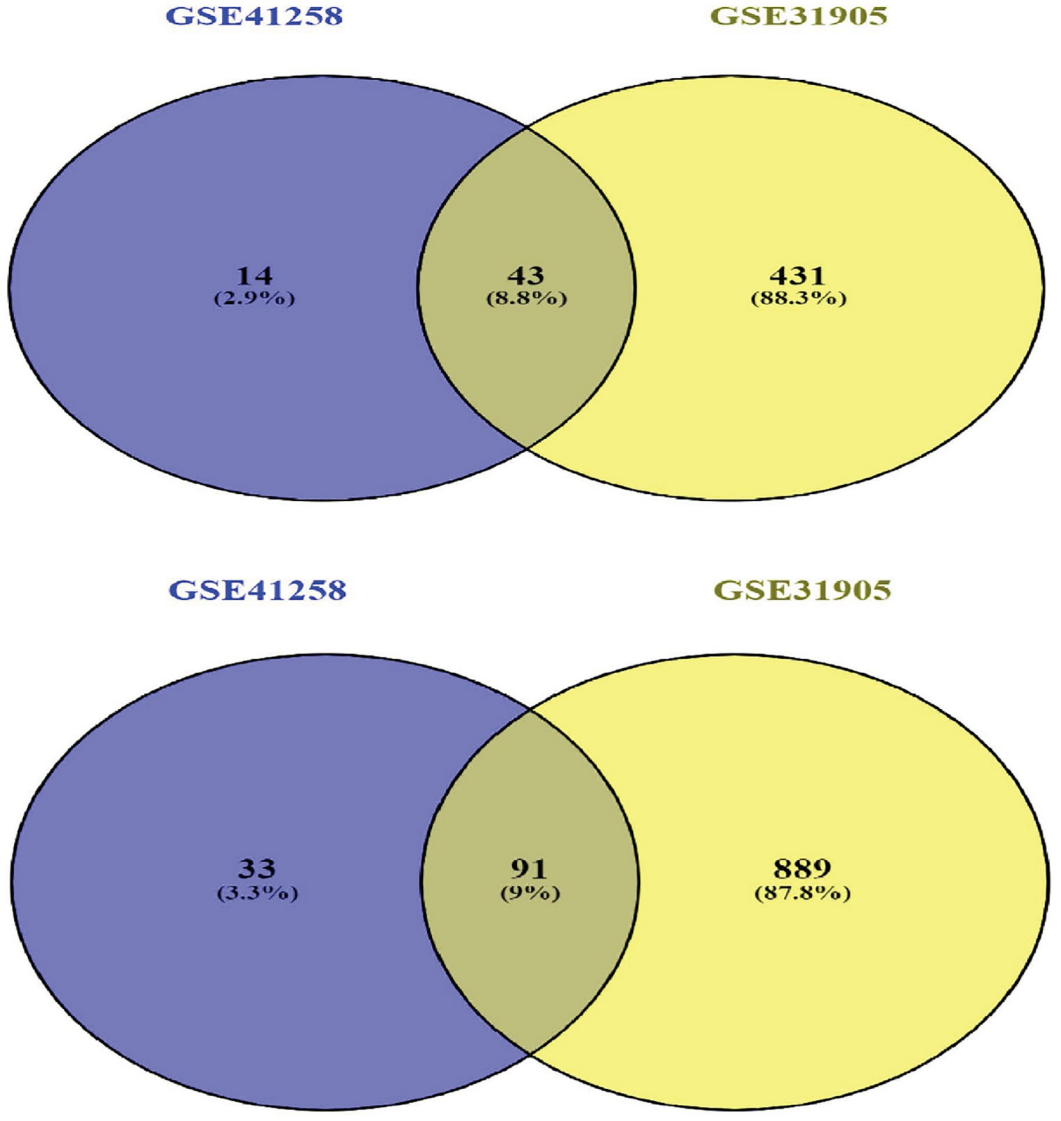
Venn diagrams illustrating the number of validated Differentially Expressed Genes (vDEGs). Top) Over-expressed DEGs. Bottom) Under-expressed DEGs.

According to our analysis of GSE41258 (Fig 1), 181 DEGs, out of which 124 and 57 were down-regulated and up-regulated respectively, were detected (S1 Table). Furthermore, the analysis of GSE101764 (Fig 2) produced 9331 DMGs, out of which 3758 and 5573 were included in hyper-methylated and hypo-methylated DMGs, respectively (S2 Table). The methylation status of samples in GSE101764 is shown in Fig 2. Additionally, the comparison between M-values and Beta-values of those samples are illustrated in Fig 3. Pursuant to the analysis of GSE31905 (Fig 2), as a validation dataset, 1454 DEGs, out of which 980 down-regulated and 474 up-regulated genes were found (S3 Table). The top-ten validated differentially expressed genes in both under-expressed and over-expressed categories are included in the Table 1. Accordingly, 134 DEGs, out of which 91 under-expressed genes to the accompaniment of 43 over-expressed genes were validated (Table 2) (Fig 4).

**Table 1:** The list of top-ten validated Differentially Expressed Genes ordered by their |LogFC| values in the discovery dataset (GSE41258).

*Over-Expressed vDEGs*			*Under-Expressed vDEGs*		
*Gene Names*	*ID*	*|LogFC|*	*Gene Names*	*ID*	*|LogFC|*
*CDH3*	*1001*	*6*.*16865195*	*GCG*	*2641*	*8*.*08903419*
*COL11A1*	*1301*	*4*.*83916367*	*PYY*	*5697*	*7*.*51876197*
*COL10A1*	*1300*	*4*.*05230173*	*CLCA4*	*22802*	*6*.*01655813*
*SLC6A6*	*6533*	*3*.*93126816*	*CA1*	*759*	*6*.*00628353*
*CHI3L1*	*1116*	*3*.*85808587*	*ZG16*	*653808*	*5*.*86412993*
*CST1*	*1469*	*3*.*62983577*	*CHP2*	*63928*	*5*.*04082639*
*INHBA*	*3624*	*3*.*51967818*	*MT1M*	*4499*	*4*.*44971744*
*MMP11*	*4320*	*3*.*40191178*	*MS4A12*	*54860*	*4*.*42473303*
*CEMIP*	*57214*	*3*.*38089498*	*CA2*	*760*	*4*.*10522872*
*EFNA3*	*1944*	*3*.*26112451*	*SLC26A3*	*1811*	*4*.*09489003*

**Table 2:** The list of Differentially Expressed Genes are in common between discovery dataset (GSE41258) and validation dataset (GSE31905).

*Over-Expressed vDEGs*				*Under-Expressed vDEGs*					
*Gene Names*	*ID*	*Gene Names*	*ID*	*Gene Names*	*ID*	*Gene Names*	*ID*	*Gene Names*	*ID*
*CDH3*	*1001*	*SERPINB5*	*5268*	*ARL14* ^*a*^	*80117*	*CLEC3B*	*7123*	*SI*	*6476*
*COL11A1*	*1301*	*TDGF1P3*	*6998*	*SRPX*	*8406*	*PDE9A*	*5152*	*SLC26A3*	*1811*
*COL10A1*	*1300*	*MMP3*	*4314*	*KLF4*	*9314*	*LGALS2*	*3957*	*CA2*	*760*
*SLC6A6*	*6533*	*THBS2*	*7058*	*KRT20*	*54474*	*MMP28*	*79148*	*MS4A12*	*54860*
*CHI3L1*	*1116*	*CXCL11*	*6373*	*PLCE1*	*51196*	*SLC26A2*	*1836*	*MT1M*	*4499*
*CST1*	*1469*	*CCL20*	*6364*	*CDH19*	*28513*	*LYVE1*	*10894*	*CHP2*	*63928*
*INHBA*	*3624*	*BGN*	*633*	*OGN*	*4969*	*ATP1A2*	*477*	*ZG16*	*653808*
*MMP11*	*4320*	*CLDN1*	*9076*	*TSPAN7*	*7102*	*JCHAIN*	*3512*	*CA1*	*759*
*CEMIP*	*57214*	*CELSR3* ^*a*^	*1951*	*TCF21*	*6943*	*CFD*	*1675*	*CLCA4*	*22802*
*EFNA3*	*1944*	*SLC7A5*	*8140*	*TNXB* ^*a*^	*7148*	*MUC2*	*4583*	*PYY*	*5697*
*MMP7*	*4316*			*EPB41L3*	*23136*	*BCHE*	*590*	*GCG*	*2641*
*ITGBL1*	*9358*			*FGFR2*	*2263*	*ADAMDEC1*	*27299*		
*CXCL8*	*3576*			*PLAC8*	*51316*	*PCK1* ^*a*^	*5105*		
*DPEP1*	*1800*			*HSD11B2*	*3291*	*CDHR5*	*53841*		
*TESC*	*54997*			*GCNT3*	*9245*	*FABP1*	*2168*		
*ETV4*	*2118*			*DHRS11*	*79154*	*FAM107A*	*11170*		
*MMP1*	*4312*			*HPGD*	*3248*	*IGH*	*3492*		
*SERPINE1*	*5054*			*METTL7A*	*25840*	*DPT*	*1805*		
*CXCL3*	*2921*			*MYH11*	*4629*	*SOSTDC1* ^*a*^	*25928*		
*KLK10*	*5655*			*NPY1R* ^*a*^	*4886*	*CA4*	*762*		
*COL1A1*	*1277*			*IGLV1-44* ^*a*^	*NA*	*AKR1B10*	*57016*		
*KRT23*	*25984*			*UGT2B15* ^*a*^	*7366*	*GUCA2A*	*2980*		
*SPP1*	*6696*			*TMEM100*	*55273*	*C7*	*730*		
*ASCL2*	*430*			*UGT1A8*	*54576*	*CXCL12*	*6387*		
*FAP*	*2191*			*HMGCS2*	*3158*	*CEACAM7*	*1087*		
*UBD*	*10537*			*IGHA2* ^*a*^	*3494*	*SLC4A4*	*8671*		
*TRIB3*	*57761*			*KCNMA1*	*3778*	*FCGBP*	*8857*		
*MSLN*	*10232*			*VIP*	*7432*	*GPM6B*	*2824*		
*TACSTD2*	*4070*			*CA12*	*771*	*CLDN8*	*9073*		
*SLCO1B3*	*28234*			*ANPEP*	*290*	*UGT2B17*	*7367*		
*LGR5*	*8549*			*MT1F*	*4494*	*AQP8*	*343*		
*CXCL1*	*2919*			*IL1R2* ^*a*^	*7850*	*PLP1*	*5354*		
*MFAP2* ^*a*^	*4237*			*LRRC19*	*64922*	*GPM6A*	*2823*		
				*P2RY14* ^*a*^	*9934*	*CLCA1*	*1179*		
				*CHRDL1*	*91851*	*UGT2A3* ^*a*^	*79799*		
				*SCNN1B*	*6338*	*ADH1C*	*126*		
				*MUC4*	*4585*	*CWH43*	*80157*		
				*HSD17B2*	*3294*	*ADH1B*	*125*		
				*NR3C2*	*4306*	*NXPE4*	*54827*		
				*DES*	*1674*	*ABCA8*	*10351*		

-The “^a^” symbols represent our novel findings in the expression profile.

### 2.2. Identification of overlapping validated Differentially Expressed Genes-Differentially Methylated Genes (vDEGs-DMGs)

**Fig 5:**
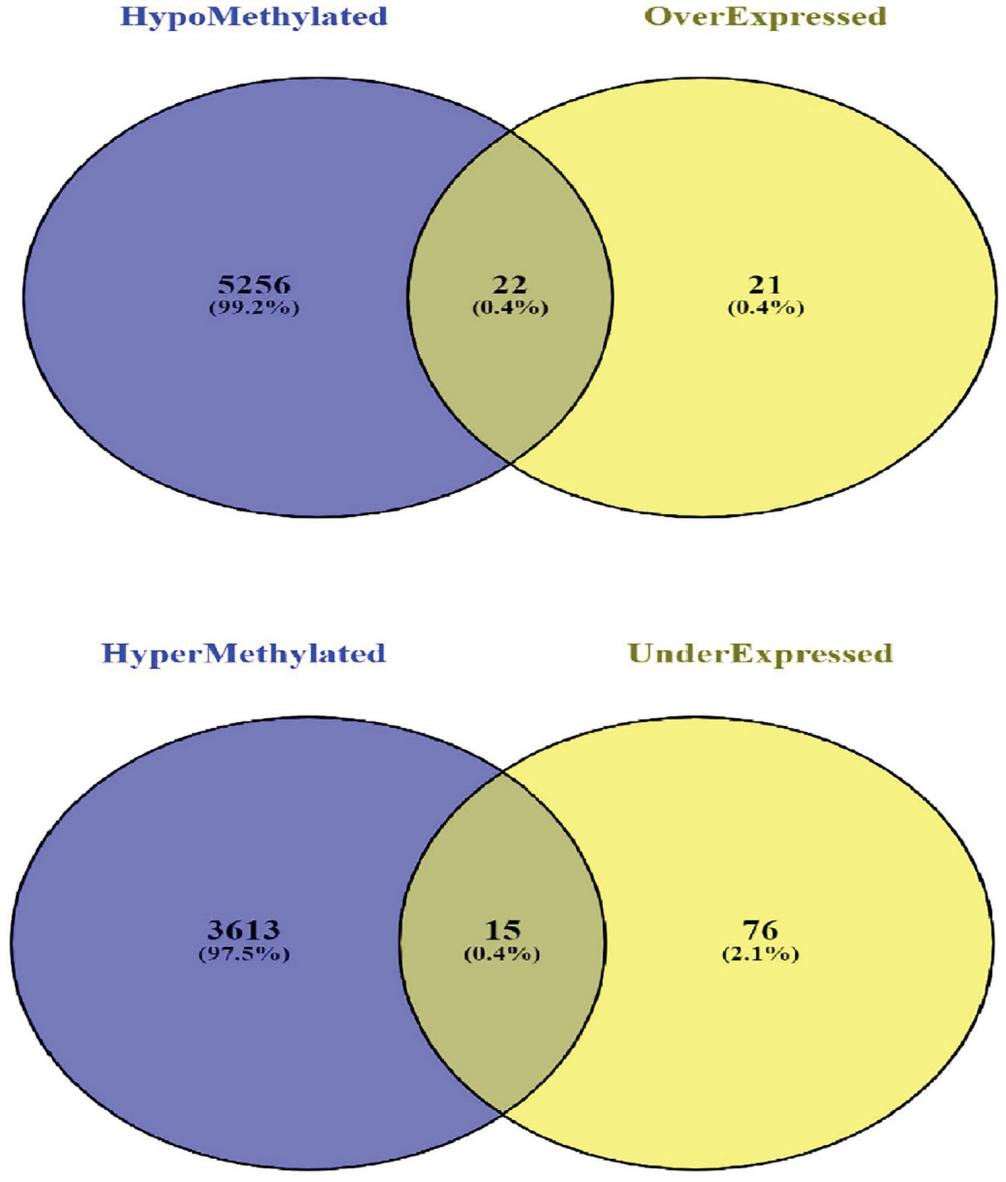
Venn diagram depicting the number of overlapped validated Differentially Expressed Genes-Differentially Methylated Genes (vDEGs-DMGs). Top) Over-expressed/hypo-methylated vDEGs-DMGs. Bottom) Under-expressed/hyper-methylated vDEGs-DMGs.

**Fig 6:**
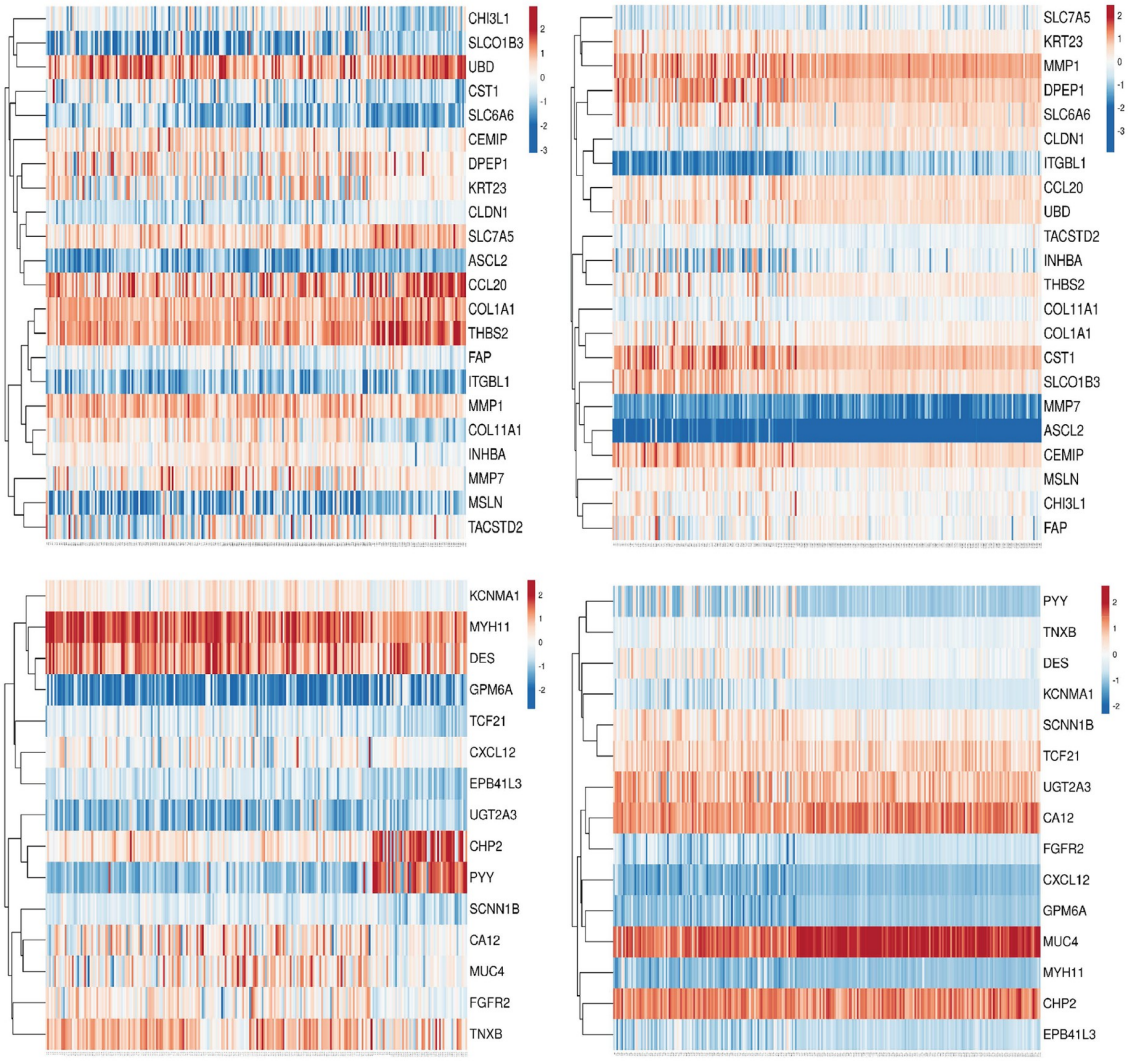
Heatmaps as for expression and methyaltion of validated-Differentially-Expressed-Genes-Differentially-Methylated-Genes (vDEGs-DMGs) based on our participants’ data. The top left corner) Expression values of over-expressed/hypo-methylated vDEGs-DMGs. The bottom left corner) Expression values of under-expressed/hyper-methylated vDEGs-DMGs. The top right corner) Methylation values of over-expressed/hypo-methylated vDEGs-DMGs. The bottom right corner) Methylation values of under-expressed/hyper-methylated vDEGs-DMGs.

**Fig 7:**
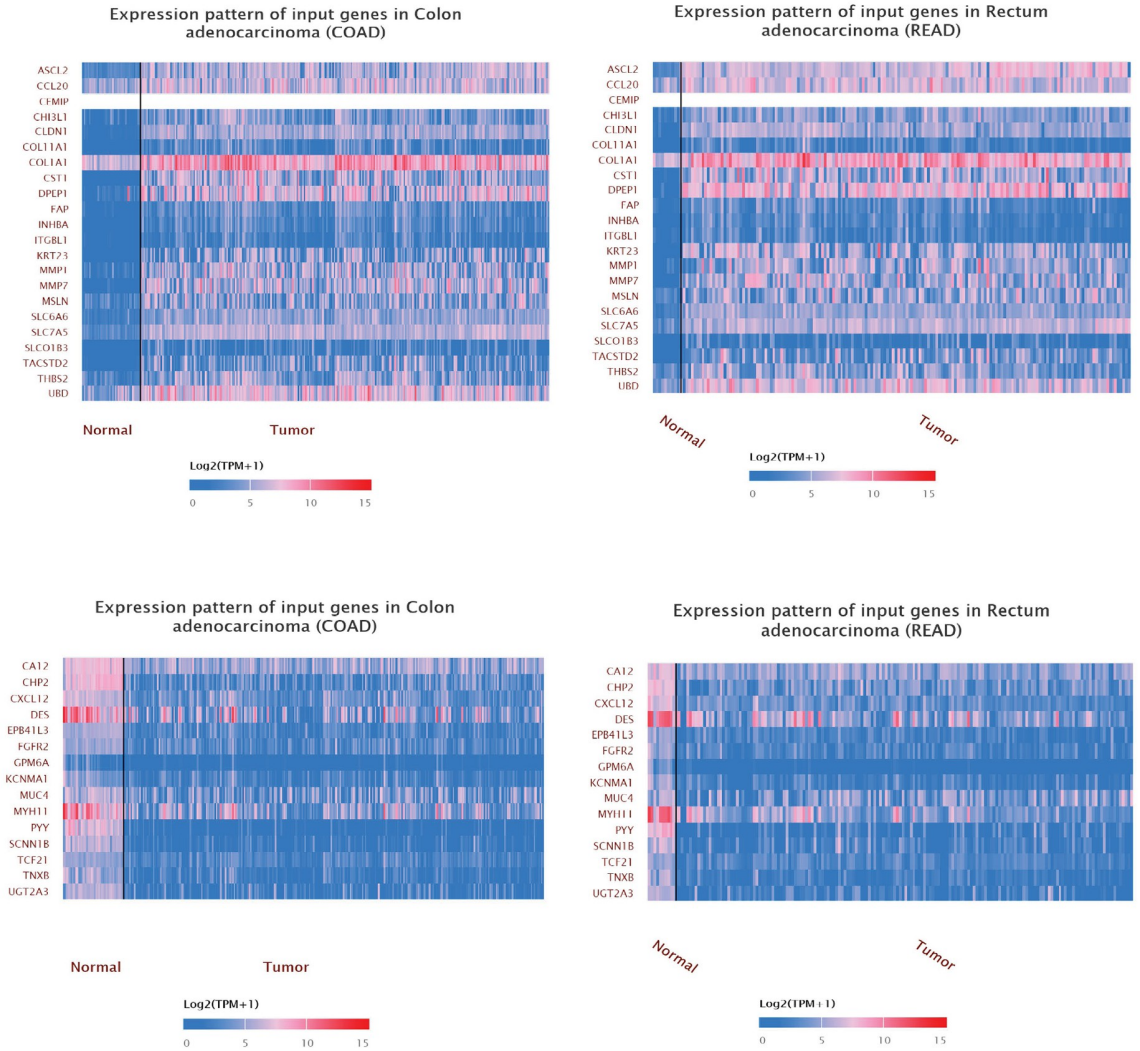
Heatmaps as for expression of validated-Differentially-Expressed-Genes-Differentially-Methylated-Genes (vDEGs-DMGs) based on TCGA data. The top left corner) Expression values of over-expressed/hypo-methylated vDEGs-DMGs in COAD. The bottom left corner) Expression values of under-expressed/hyper-methylated vDEGs-DMGs in COAD. The top right corner) Expression values of over-expressed/hypo-methylated vDEGs-DMGs in READ. The bottom right corner) Expression values of under-expressed/hyper-methylated vDEGs-DMGs in READ.

Among all vDEGs and DMGs, 22 hypo-methylated/over-expressed genes (ASCL2, CCL20, CEMIP, CHI3L1, CLDN1, COL11A1, COL1A1, CST1, DPEP1, FAP, INHBA, ITGBL1, KRT23, MMP1, MMP7, MSLN, SLC6A6, SLC7A5, SLCO1B3, TACSTD2, THBS2, and UBD) and 15 hyper-methylated/under-expressed genes (CA12, CHP2, CXCL12, DES, EPB41L3, FGFR2, GPM6A, KCNMA1, MUC4, MYH11, PYY, SCNN1B, TCF21, TNXB, and UGT2A3) were found to be overlapped (Fig 5). The heatmap plots for these vDEGs-DMGs are depicted based on their expression and methylation values in Figs 6 & 7.

### 2.3. Verification of Differentially Methylated Genes (DMGs) based on MEXPRESS

**Fig 8:**
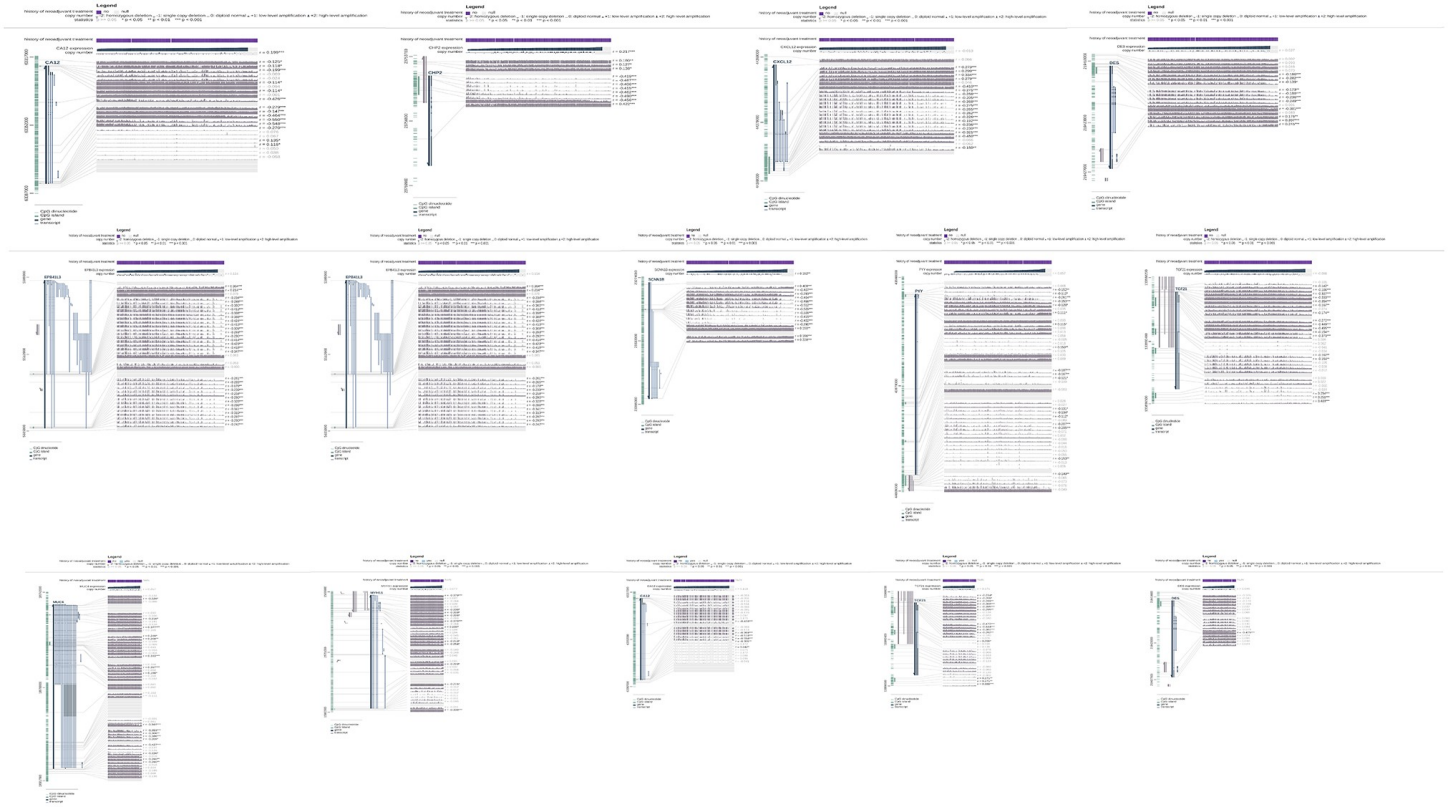
MEXPRESS results of statistically significant under-expressed/hyper-methylated validated Differentially Expressed Genes-Differentially Methylated Genes (vDEGs-DMGs). The first nine results pertain to COAD cancer type and the last five results correspond to READ cancer type.

**Fig 9:**
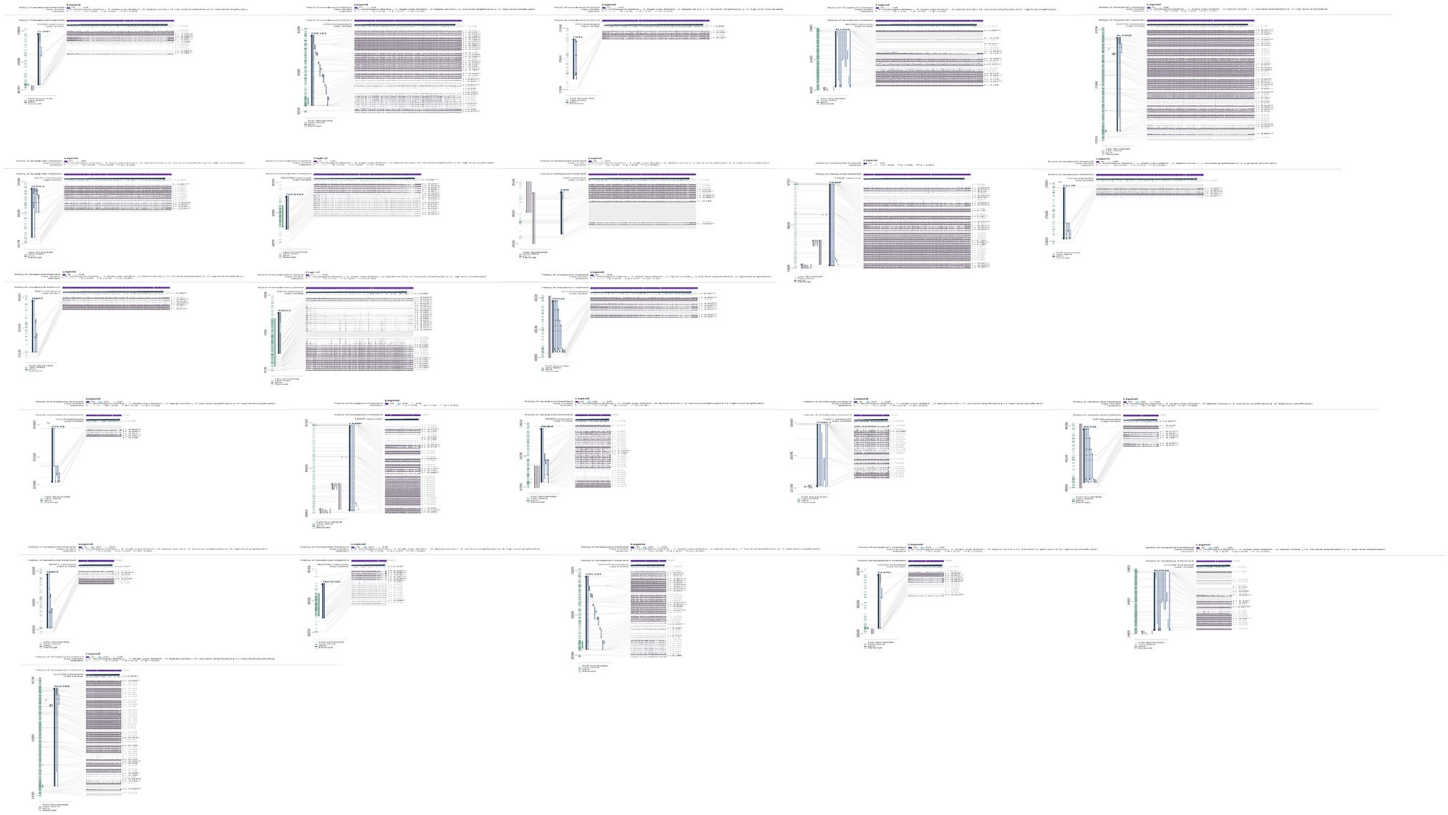
MEXPRESS results of statistically significant over-expressed/hypo-methylated validated Differentially Expressed Genes-Differentially Methylated Genes (vDEGs-DMGs). The first thirteen results pertain to COAD cancer type and the last eleven results correspond to READ cancer type.

The results produced by the MEXPRESS demonstrated nine and five significant hyper-methylated/under-expressed genes in COAD and READ cancer types, respectively (Fig 8). By the same token, 13 and 11 significant hypo-methylated/over-expressed genes were observed in COAD and READ cancer types, respectively (Fig 9). Also, we had 16 non-significant hyper-methylated/under-expressed genes and 20 non-significant hypo-methylated/over-expressed genes in both cancer types in total. Considering the r-value in the Figs 8 and 9, it indicates the quantity and type (positive or negative) of correlations between the methylation and expression status of various probes on each gene. The determining criterion that we considered to include the significant genes was the remarkable higher numbers of negative r-values in comparison with positive ones in each gene demonstrating more confident negative correlation between the methylation and expression status on a specific gene.

### 2.4. Scrutiny of negative correlation between expression and methylation status with correlation plot

**Fig 10:**
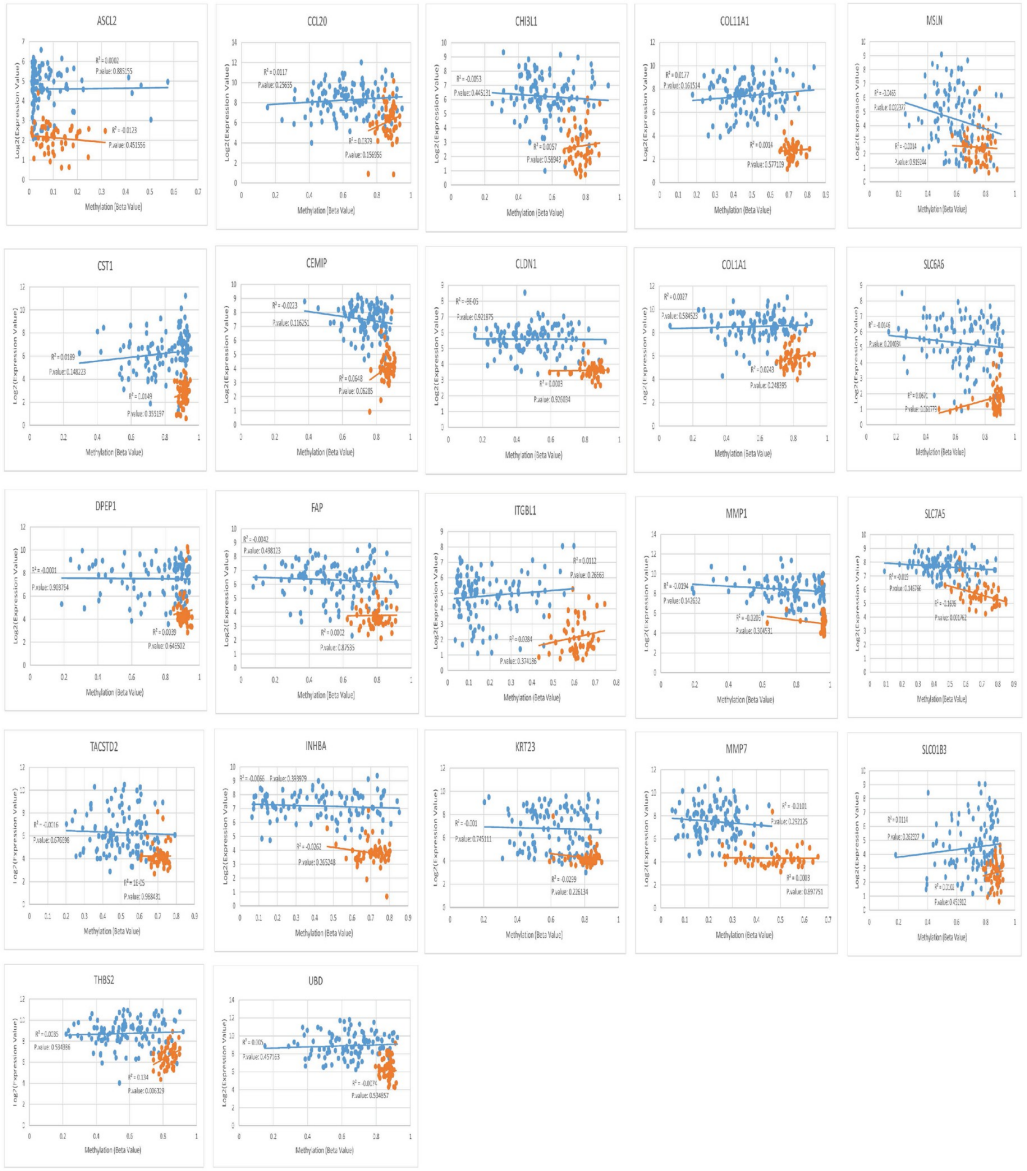
Correlation plots as for over-expressed/hypo-methylated validated Differentially Expressed Genes-Differentially Methylated Genes (vDEGs-DMGs).

**Fig 11:**
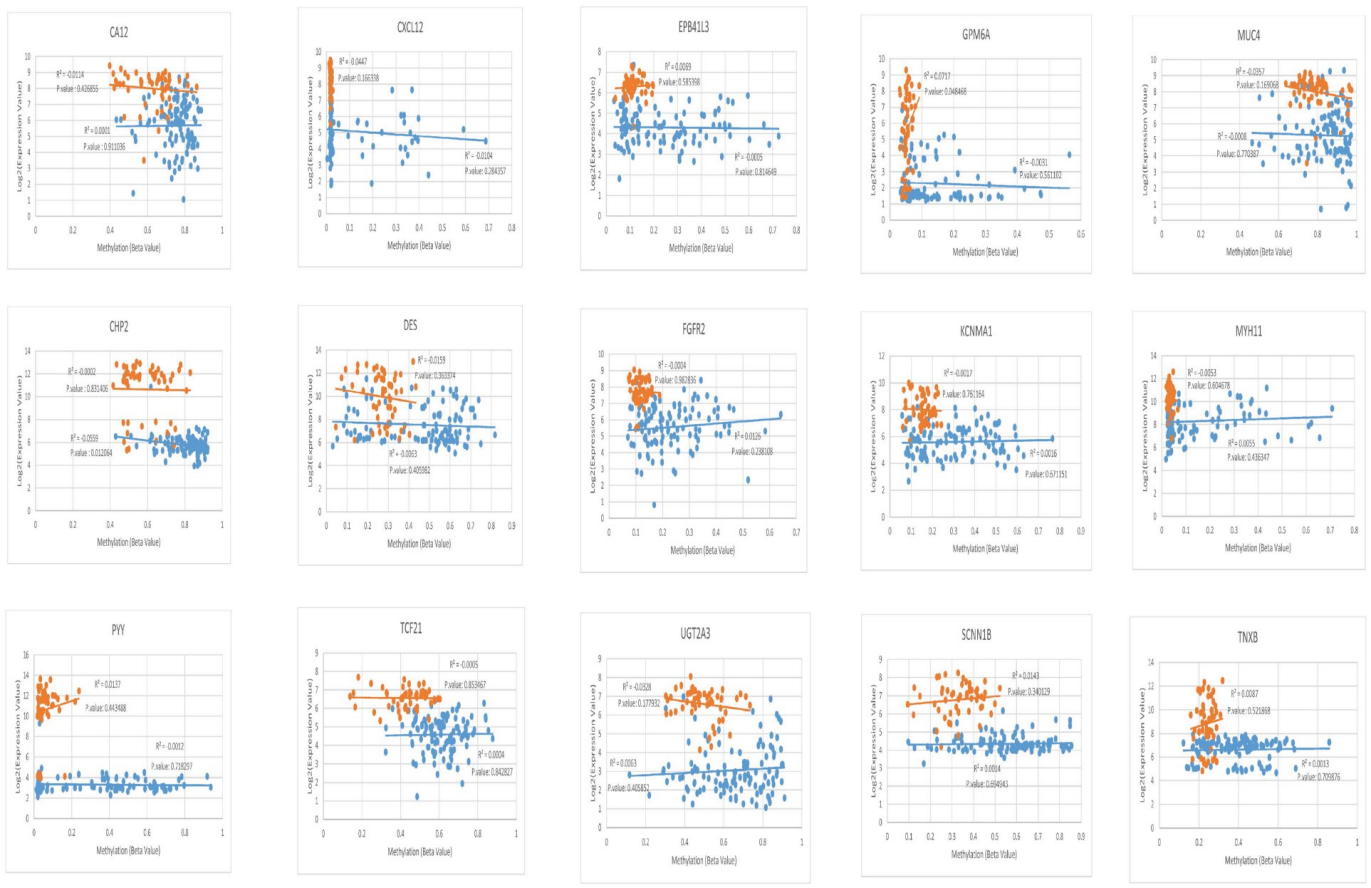
Correlation plots as for under-expressed/hyper-methylated validated Differentially Expressed Genes-Differentially Methylated Genes (vDEGs-DMGs).

Out of all correlation plots drawn for vDEGs-DMGs, solely two genes had statistically significant inverse/negative correlations between their expression and methylation status. CHP2, a hyper-methylated/under-expressed gene, and MSLN, a hypo-methylated/over-expressed gene, were our findings in this regard. The correlation plots of vDEGs-DMGs are illustrated in Figs 10 & 11.

### 2.5. Determination of specificity and sensitivity using receiver operating characteristic curves

**Fig 12:**
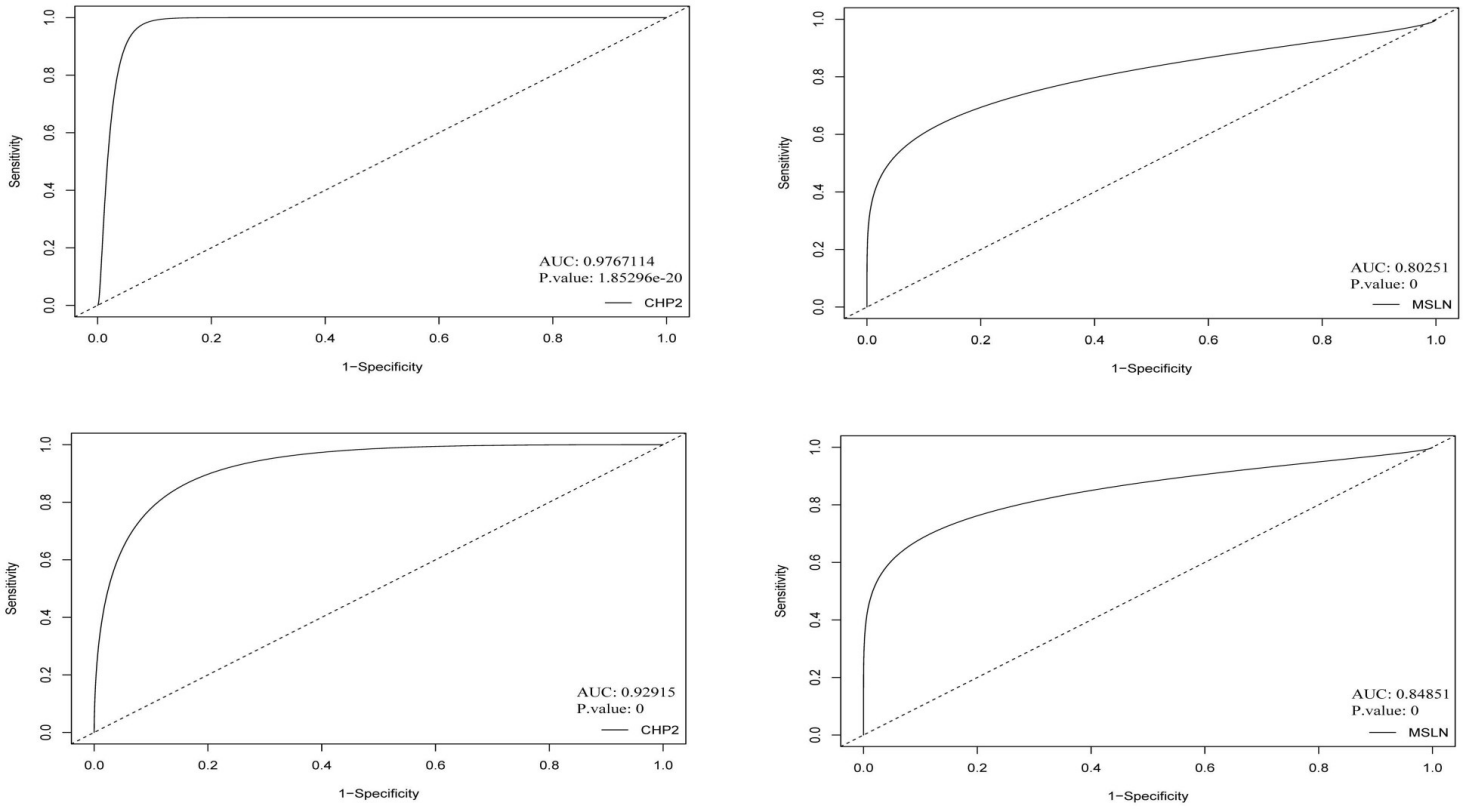
ROC curves for novel validated Differentially Expressed Genes-Differentially Methylated Genes (vDEGs-DMGs). The top curves relate to expression and the bottom curves pertain to methylation.

**Fig 13:**
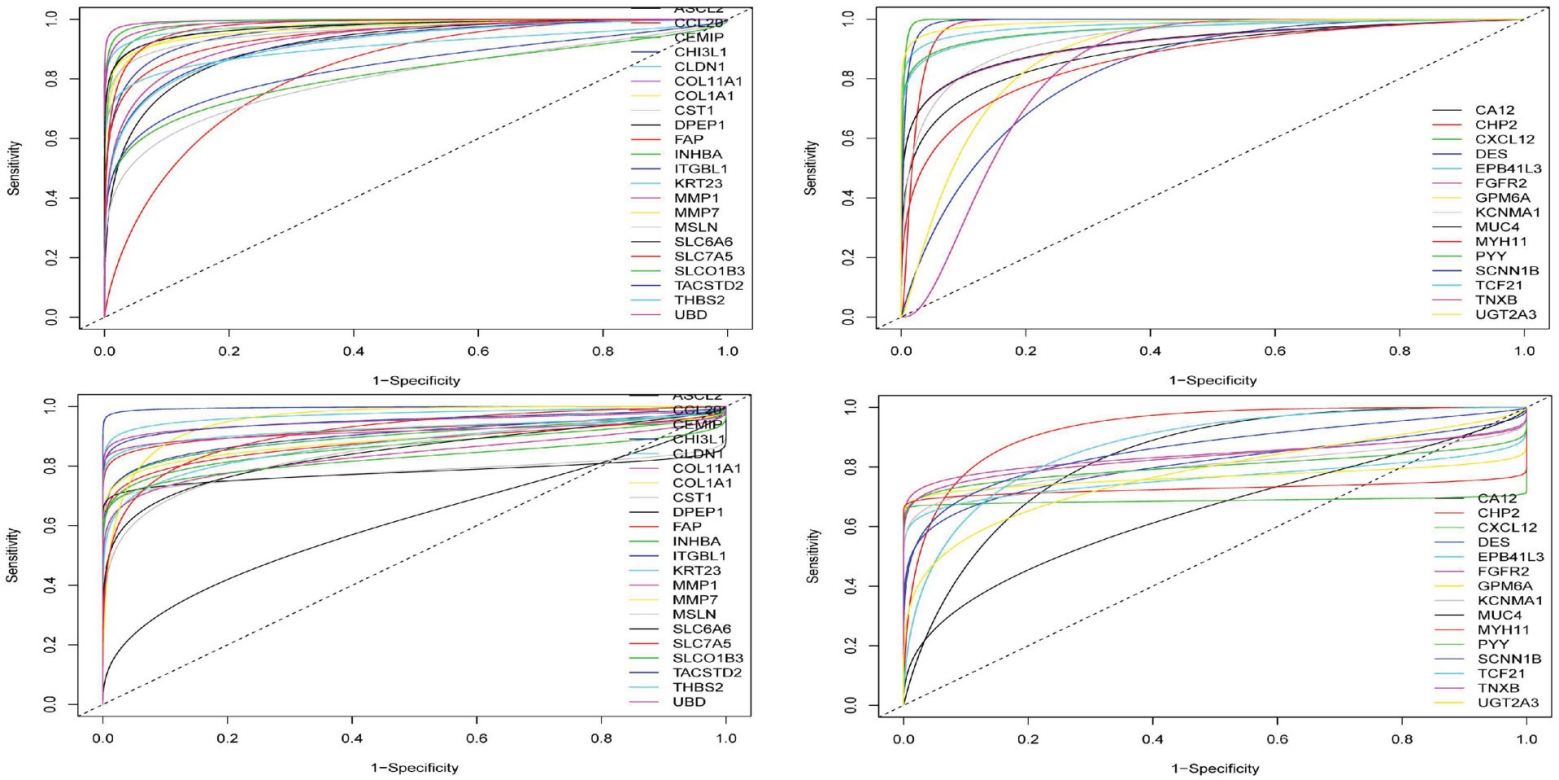
ROC curves for known validated Differentially Expressed Genes-Differentially Methylated Genes (vDEGs-DMGs). The top curves) Expression profiles. The bottom curves) methylation profiles. The left curves) Over-expressed/hypo-methylated vDEGs-DMGs. The right curves) Under-expressed/hyper-methylated vDEGs-DMGs.

Based on the expression values and the methylation values, the ROC curves assisted us in determining the specificity and the sensitivity of the inverse/negative correlations between the expression and methylation status in CHP2 and MSLN genes which had been previously confirmed (Fig 12). Taking the AUC and P.values into account, the ROC curves showed that CHP2 and MSLN genes can play role as high-quality markers in colorectal cancer. The similar ROC curves as for other vDEGs-DMGs are supplied in Fig 13. The AUC and P.values pertaining to other vDEGs-DMGs are in Table 3 in order to present them in a recognizable style.

**Table 3:** The area under curves as for vDEGs-DMGs to the accompaniment of their statistically significance.

*ROC Curve Values as for Expression Profile of Over-Expressed vDEGs-DMGs*			*ROC Curve Values as for Expression Profile of Under-Expressed vDEGs-DMGs*			*ROC Curve Values as for Methylation Profile of Hypo-Methylated vDEGs-DMGs*			*ROC Curve Values as for Methylation Profile of Hyper-Methylated vDEGs-DMGs*		
*Marker*	*AUC*	*P-value*	*Marker*	*AUC*	*P-value*	*Marker*	*AUC*	*P-value*	*Marker*	*AUC*	*P-value*
*ASCL2*	*0*.*97535*	*0*	*CA12*	*0*.*89168*	*0*	*ASCL2*	*0*.*6122*	*0*.*0211*	*CA12*	*0*.*83041*	*0*
*CCL20*	*0*.*82567*	*0*	*CHP2* ^*a*^	*0*.*97671*	*0*	*CCL20*	*0*.*92165*	*0*	*CHP2* ^*a*^	*0*.*92915*	*0*
*CEMIP*	*0*.*98666*	*0*	*CXCL12*	*0*.*96625*	*0*	*CEMIP*	*0*.*89817*	*0*	*CXCL12*	*0*.*68697*	*0*.*00025*
*CHI3L1*	*0*.*96032*	*0*	*DES*	*0*.*82882*	*0*	*CHI3L1*	*0*.*90505*	*0*	*DES*	*0*.*80666*	*0*
*CLDN1*	*0*.*98038*	*0*	*EPB41L3*	*0*.*97941*	*0*	*CLDN1*	*0*.*97282*	*0*	*EPB41L3*	*0*.*76137*	*0*
*COL11A1*	*0*.*99671*	*0*	*FGFR2*	*0*.*91787*	*0*	*COL11A1*	*0*.*95008*	*0*	*FGFR2*	*0*.*83895*	*0*
*COL1A1*	*0*.*97395*	*0*	*GPM6A*	*0*.*88472*	*0*	*COL1A1*	*0*.*88356*	*0*	*GPM6A*	*0*.*7656*	*0*
*CST1*	*0*.*9597*	*0*	*KCNMA1*	*0*.*93598*	*0*	*CST1*	*0*.*78257*	*0*	*KCNMA1*	*0*.*80293*	*0*
*DPEP1*	*0*.*91648*	*0*	*MUC4*	*0*.*92014*	*0*	*DPEP1*	*0*.*77917*	*0*	*MUC4*	*0*.*64686*	*0*.*00192*
*FAP*	*0*.*94624*	*0*	*MYH11*	*0*.*86706*	*0*	*FAP*	*0*.*87828*	*0*	*MYH11*	*0*.*72791*	*0*
*INHBA*	*0*.*99456*	*0*	*PYY*	*0*.*99671*	*0*	*INHBA*	*0*.*86494*	*0*	*PYY*	*0*.*80064*	*0*
*ITGBL1*	*0*.*92044*	*0*	*SCNN1B*	*0*.*99264*	*0*	*ITGBL1*	*0*.*99653*	*0*	*SCNN1B*	*0*.*85913*	*0*
*KRT23*	*0*.*90799*	*0*	*TCF21*	*0*.*96444*	*0*	*KRT23*	*0*.*87099*	*0*	*TCF21*	*0*.*8722*	*0*
*MMP1*	*0*.*99019*	*0*	*TNXB*	*0*.*83802*	*0*	*MMP1*	*0*.*83601*	*0*	*TNXB*	*0*.*83186*	*0*
*MMP7*	*0*.*9685*	*0*	*UGT2A3*	*0*.*98941*	*0*	*MMP7*	*0*.*95681*	*0*	*UGT2A3*	*0*.*77412*	*0*
*MSLN* ^*a*^	*0*.*80251*	*0*				*MSLN* ^*a*^	*0*.*84851*	*0*			
*SLC6A6*	*0*.*97474*	*0*				*SLC6A6*	*0*.*84332*	*0*			
*SLC7A5*	*0*.*9779*	*0*				*SLC7A5*	*0*.*91281*	*0*			
*SLCO1B3*	*0*.*81415*	*0*				*SLCO1B3*	*0*.*8251*	*0*			
*TACSTD2*	*0*.*83922*	*0*				*TACSTD2*	*0*.*95164*	*0*			
*THBS2*	*0*.*91633*	*0*				*THBS2*	*0*.*92517*	*0*			
*UBD*	*0*.*93439*	*0*				*UBD*	*0*.*91535*	*0*			

-The “^a^” symbols represent our novel findings in the expression profile.

### 2.6. Recognition of Differentially Expressed MiRNAs (DEMs) using the dbDEMC2

**Fig 14:**
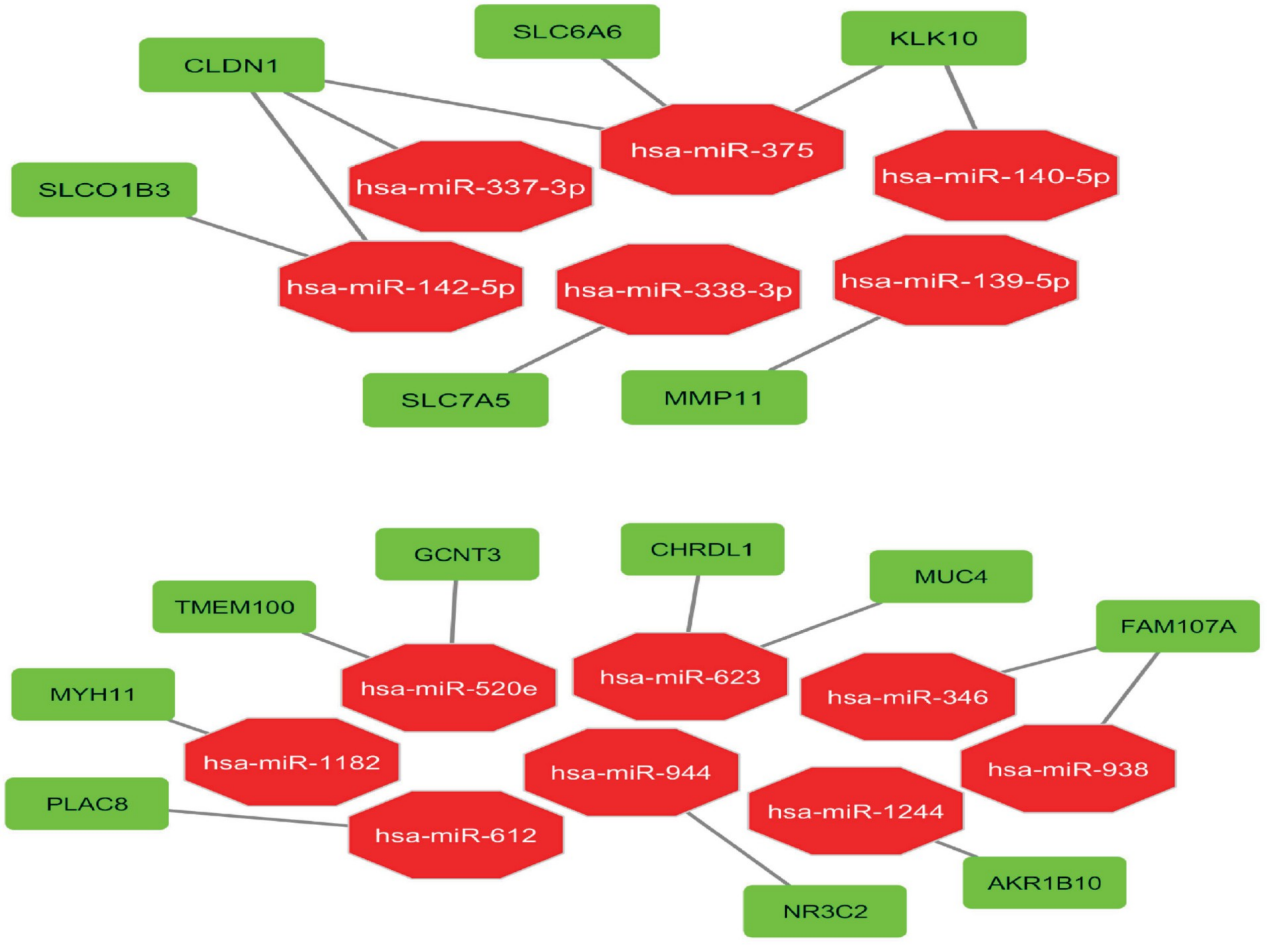
Interactions between Differentially Expressed miRNAs and their target validated Differentially Expressed Genes (vDEGs-DEMs). Top) Over-expressed vDEGs- down-regulated DEMs. Bottom) Under-expressed vDEGs- up-regulated DEMs.

79 DEMs, out of which 38 up-regulated and 41 down-regulated miRNAs were detected (S4 Table). Moreover, the interactions depicting how the found vDEGs are possibly under the regulatory control of DEMs are shown in Fig 14.

### 2.7. GEPIA-verification of validated Differentially Expressed Genes

**Fig 15:**
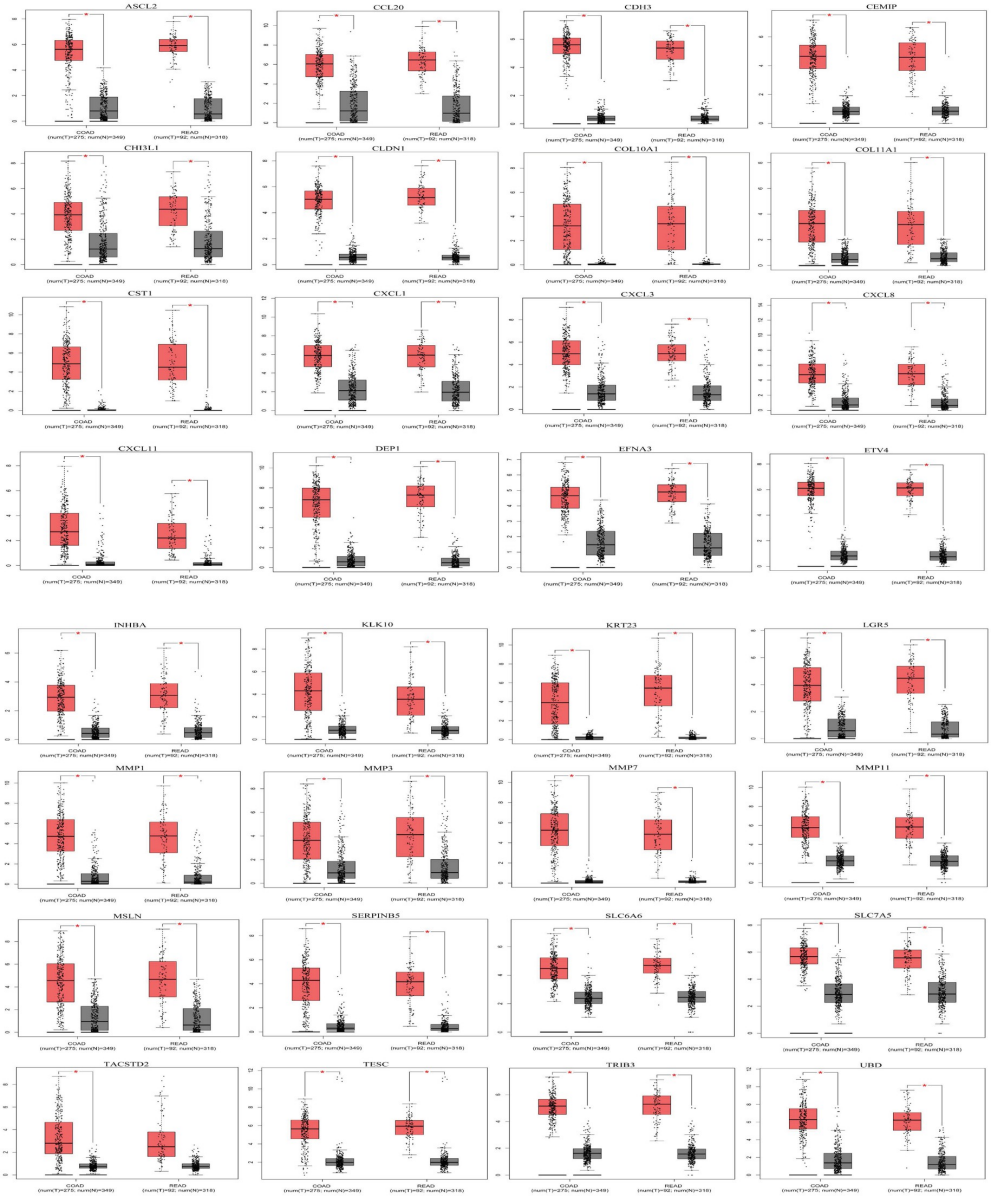
Expression box plots of statistically significant over-expressed validated Differentially Expressed Genes based on TCGA data.

**Fig 16:**
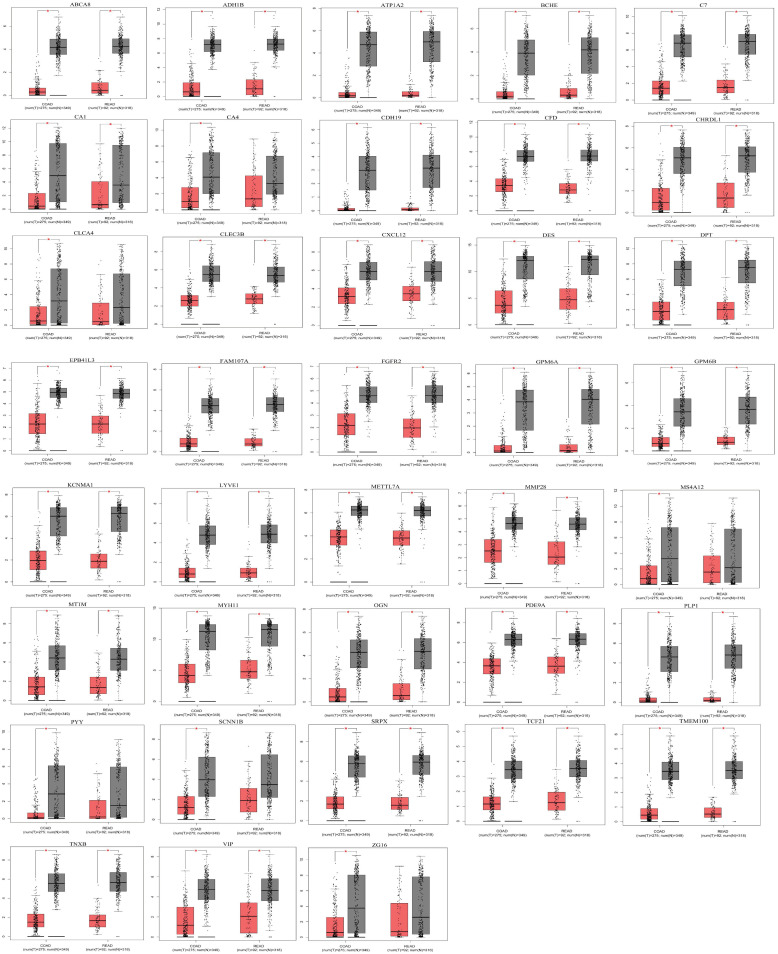
Expression box plots of statistically significant under-expressed validated Differentially Expressed Genes based on TCGA data.

The expression changes of 32 and 31 over-expressed vDEGs were verified in COAD and READ cancer types, respectively (Fig 15). Also, the GEPIA confirmed the expression change of 38 and 32 under-expressed vDEGs in COAD and READ cancer types, respectively (Fig 16).

### 2.8. Protein-protein interactions and determination of hub genes

**Fig 17:**
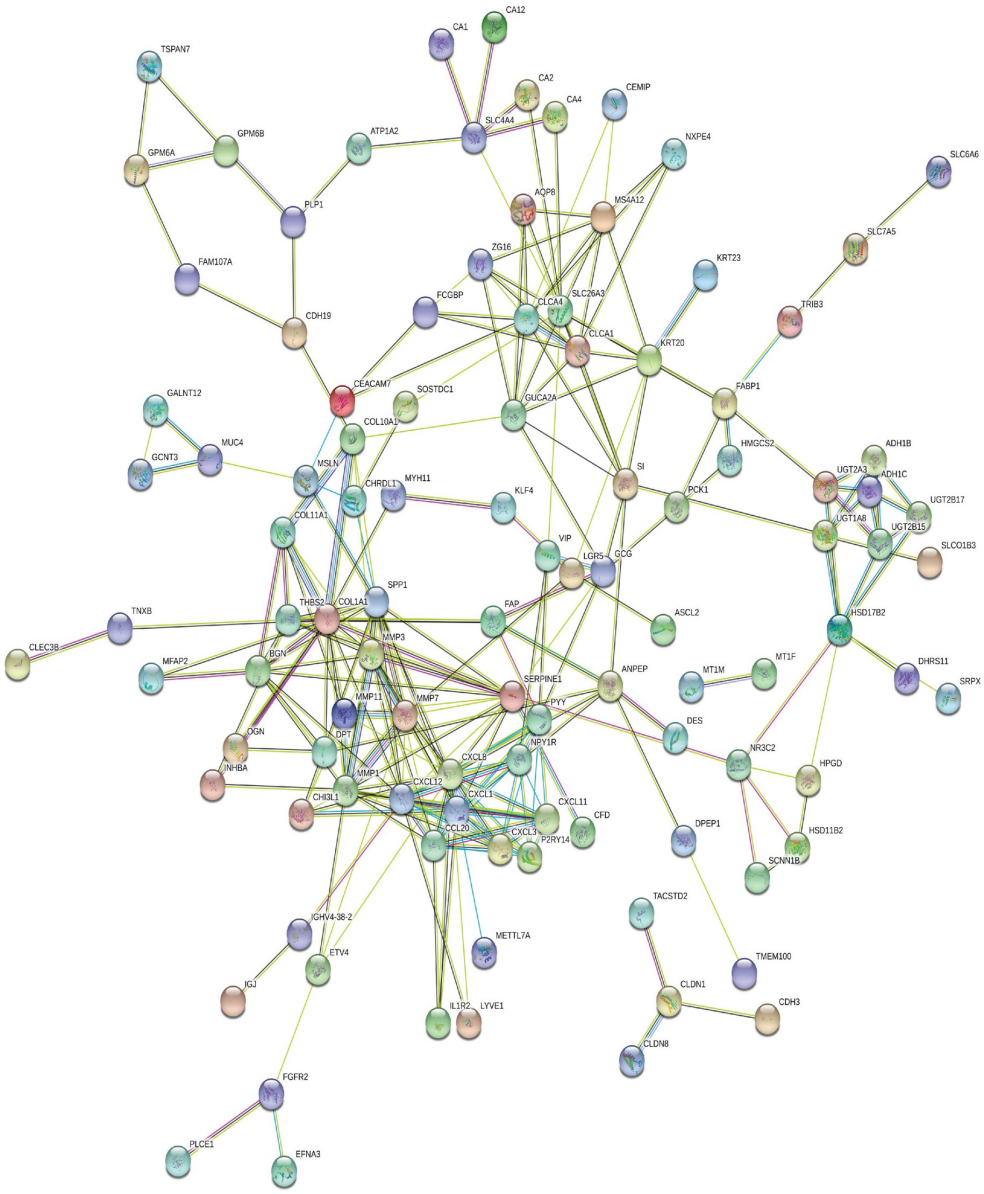
Protein protein interactions network based on the STRING database.

**Fig 18:**
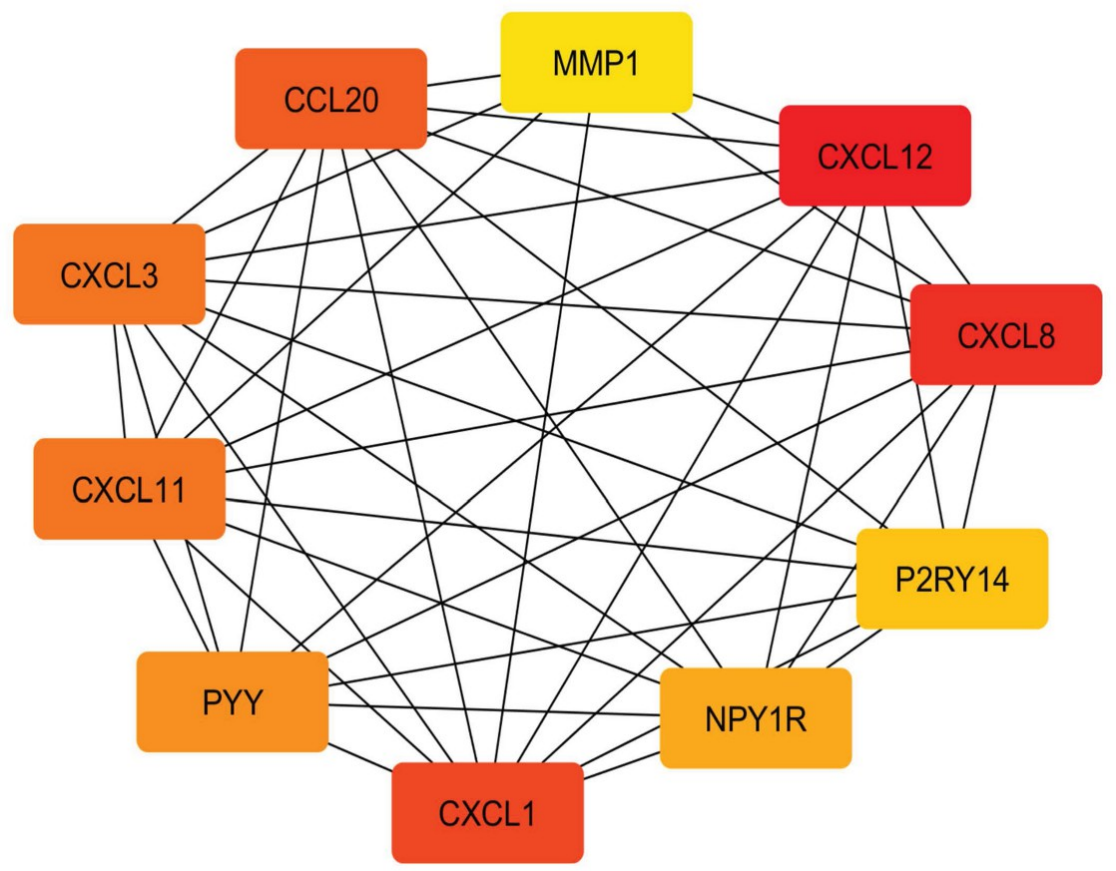
Protein protein interactions network connecting top-ten proteins based on the MCC criterion in the cyto hubba plugin.

As shown in Fig 17, the interactions connecting vDEGs-related proteins are produced based on the STRING database. To be more meticulous, the top ten hub genes having been filtered by the Cytohubba plugin are illustrated in Fig 18.

### 2.9. Functional enrichment analysis and KEGG pathways

**Fig 19:**
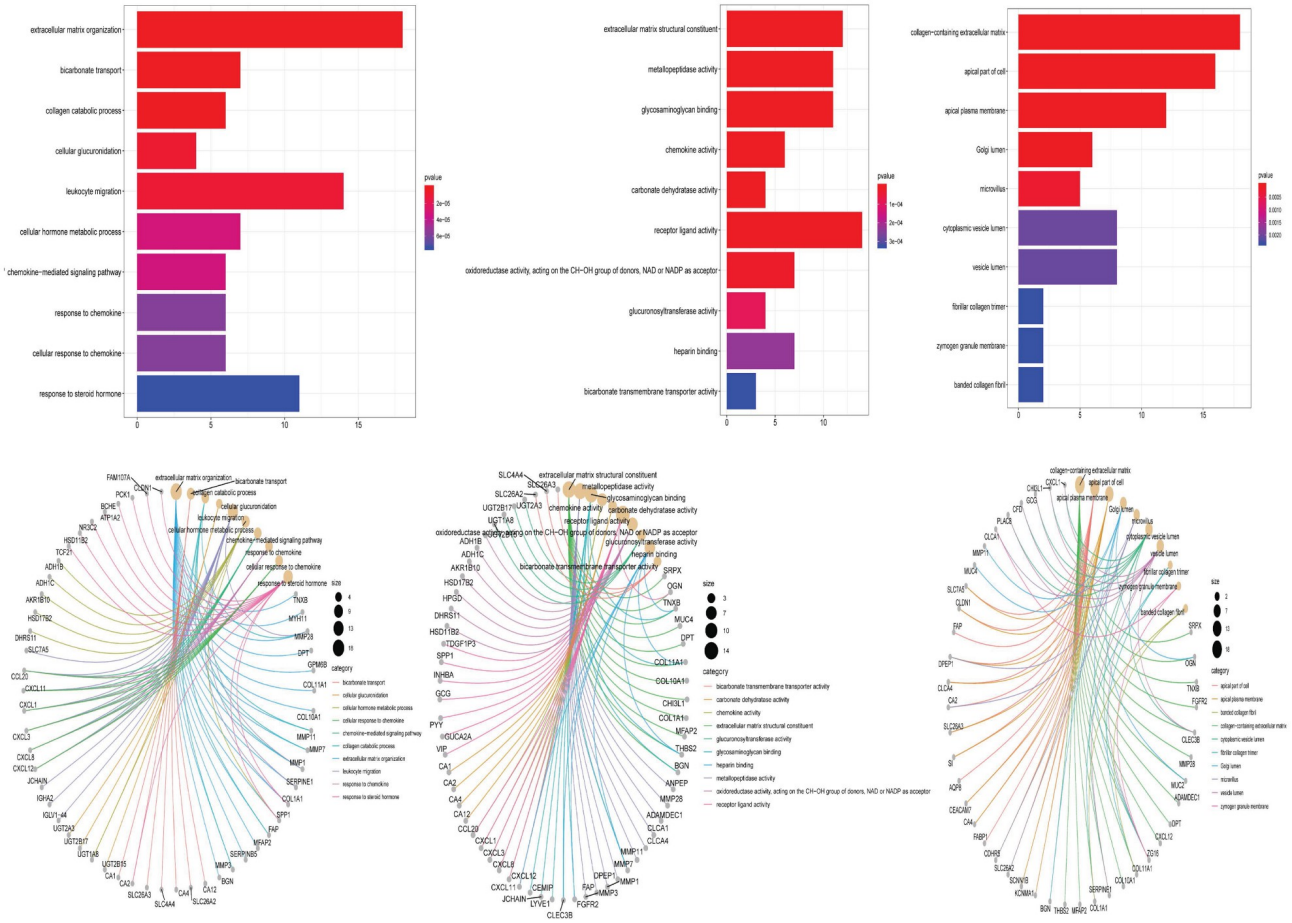
Functional enrichment analysis box plots and cnet plots. The left collumn) Biological Processes. The middle collumn) Molecular Functions. The right collumn) Cellular Components.

**Fig 20:**
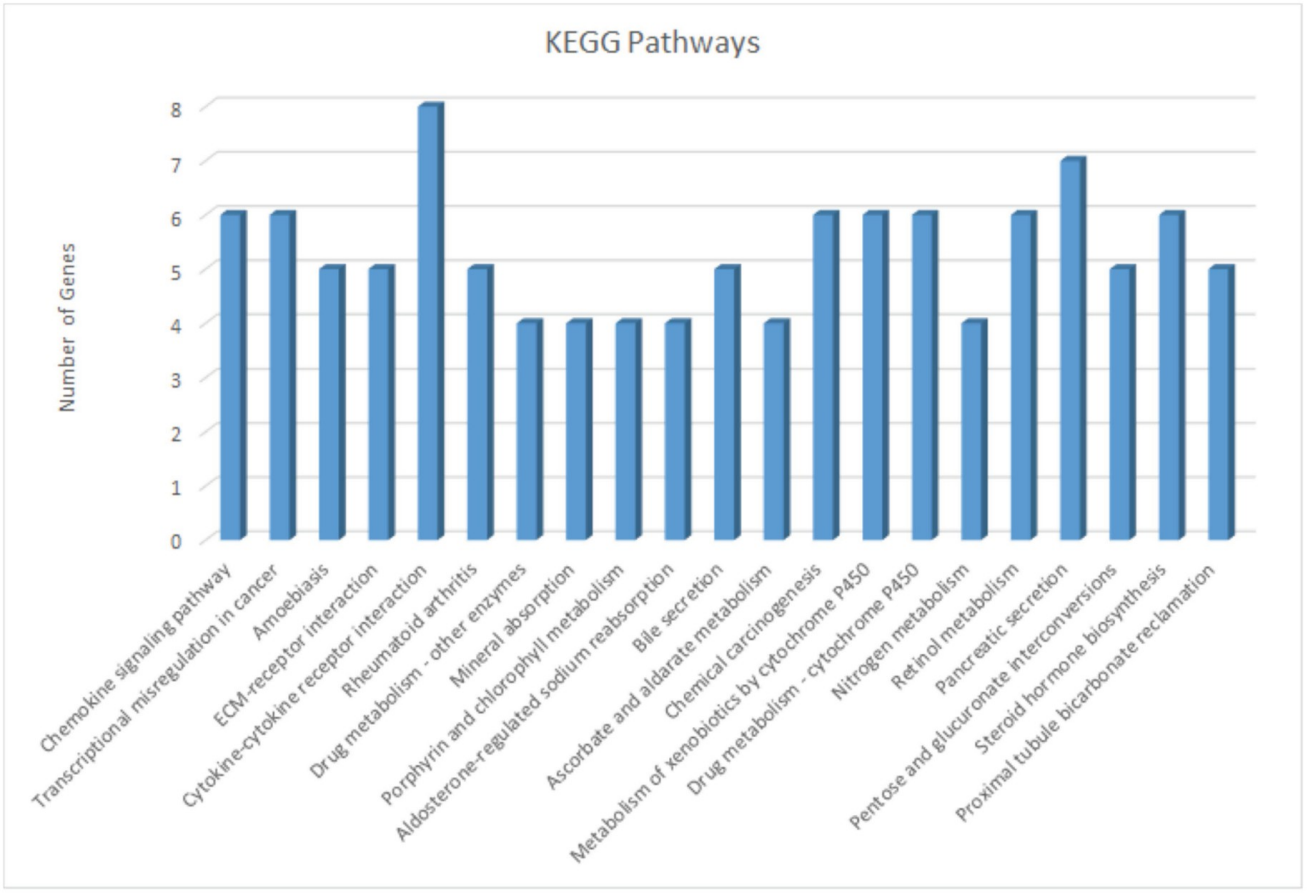
KEGG pathways of involved validated Differentially Expressed Genes (vDEGs).

The gene-ontology analysis of vDEGs produced three terms in the context of biological processes, molecular functions, and cellular components which are depicted in two styles of boxplot and cnet plot (Fig 19). Extracellular matrix organization, receptor ligand activity, and collagen−containing extracellular matrix are BP, MF, and CC with the most participated genes, respectively. Also, extracellular matrix organization, extracellular matrix structural constituent, and collagen−containing extracellular matrix are the most statistically significant BP, MF, and CC, respectively. Cytokine-cytokine receptor interaction, and pancreatic secretion are two KEGG pathways with the most participated genes (Fig 20).

### 2.10. Prognostic analysis of validated Differentially Expressed Genes (vDEGs) using Kaplan-Meier plots

**Fig 21:**
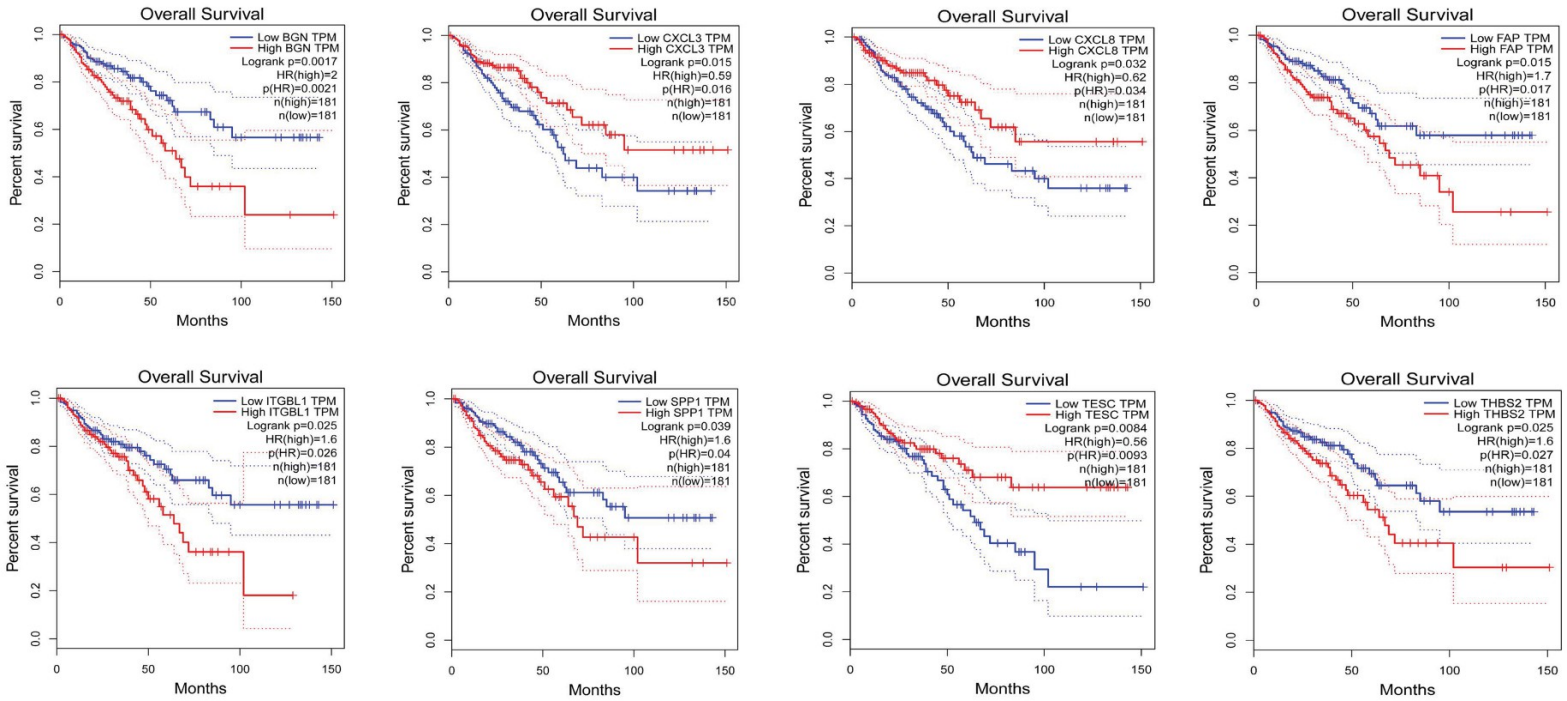
Kaplan-Meier plots of statistically significant over-expressed validated Differentially Expressed Genes. Each pale blue line corresponds to low expression in COAD or READ cancer types. Each pale red line corresponds to high expression in COAD or READ cancer types. The bold blue line represents some kind of average of low expression in two cancer types. The bold red line represents some sort of average of high expression in two cancer types.

**Fig 22:**
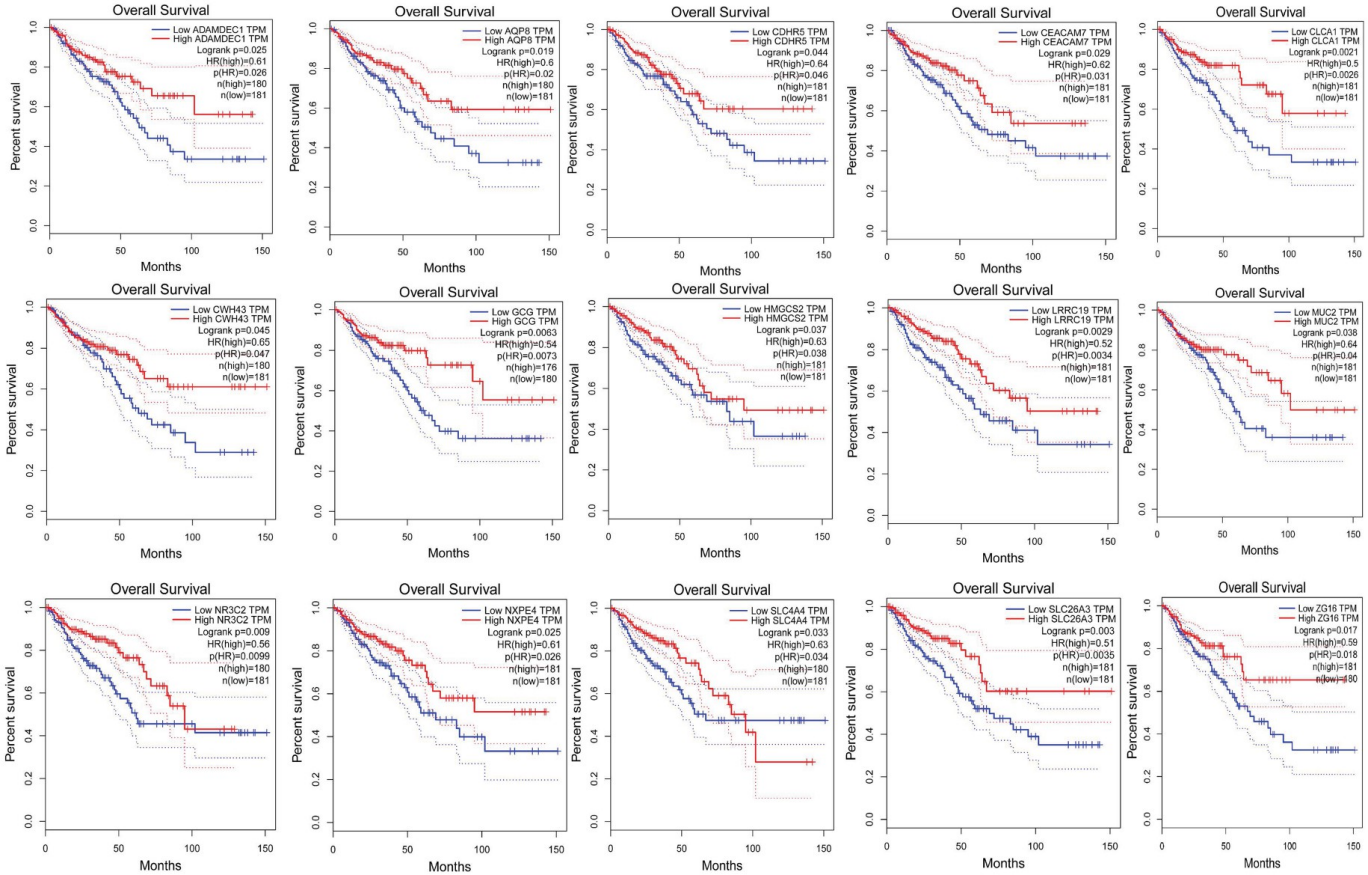
Kaplan-Meier plots of statistically significant under-expressed validated Differentially Expressed Genes. Each pale blue line corresponds to low expression in COAD or READ cancer types. Each pale red line corresponds to high expression in COAD or READ cancer types. The bold blue line represents some kind of average of low expression in two cancer types. The bold red line represents some sort of average of high expression in two cancer types.

This step of the analysis showed that eight over-expressed vDEGs to the accompaniment of 15 under-expressed vDEGs possess statistically significant prognostic value (Figs 21 & 22).

## 3. Discussion

CRC is the second cancer-related cause of death in the world [15, 23]. Since there is widespread molecular heterogeneity in CRC various clinical properties can be detected in patients. Accordingly, the routine tests such as histology cannot individually afford these complexities, and molecular markers, the information of which have been gathered in several databases and repositories, are at play [15, 24–29]. Even though the methylation process plays an important role in controlling gene expression in normal circumstances, the disruption of this phenomenon is usually recognized in approximately all malignancies [5]. Methylation modulates gene expression as for cell proliferation and differentiation. Ergo, methylation is engaged in controlling normal cell growth and cancerous cell development [5].

In the current study, we funded an attempt to establish a flowchart through using a bioinformatics approach to arrive at profiles that includes genes being distinct in their methylation and/or expression pattern.

**Fig 23:**
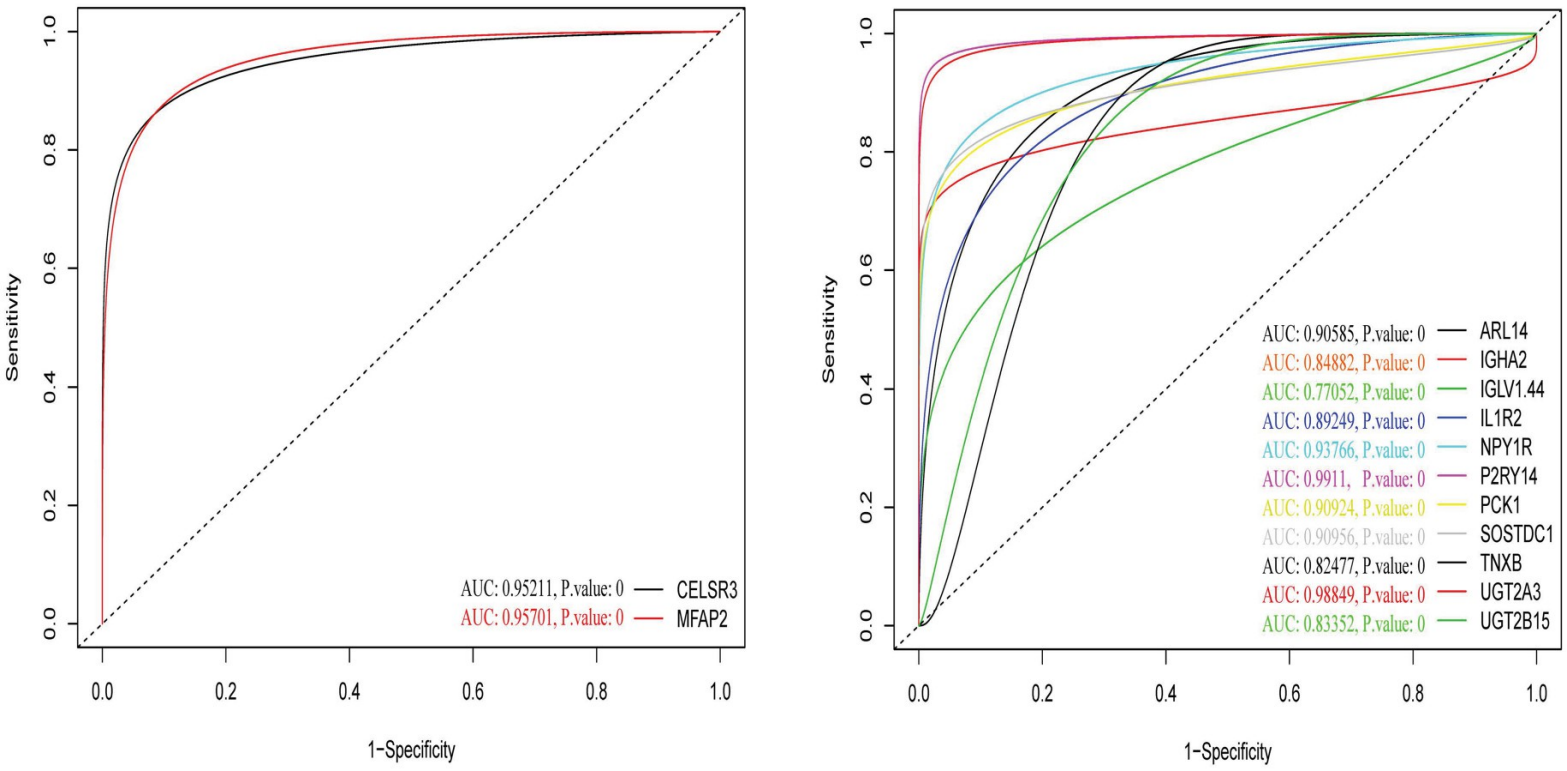
ROC curves for novel validated Differentially Expressed Genes (vDEGs). Left curve) Over-expressed novel vDEGs. Right curve) Under-expressed novel vDEGs.

Out of the detected hub genes, six (CCL20, CXCL1, CXCL3, CXCL8, CXCL11, and MMP1) were among over-expressed vDEGs which have been corroborated by the GEPIA in both COAD and READ cancer types. However, only CXCL12 was one of the hub genes whose presence in both under-expressed vDEGs and the GEPIA (COAD, and READ) has been verified. The three remaining hub genes (P2RY14, NPY1R, and PYY) were solely validated by the validation dataset. Two of the last-mentioned hub genes (P2RY14, and NPY1R), to the best of our knowledge, have not already been reported as being under-expressed in colorectal cancer. By the same token, three genes (CXCL3, CXCL8, and TESC) not only have been their diagnostic values confirmed by the validation dataset and the GEPIA (COAD, and READ) but also, their prognostic values are proved by the Kaplan-Meier plots. Accordingly, five hub genes (CCL20, CXCL1, CXCL11, CXCL12, and MMP1) are advantageous solely in diagnosis as being completely verified (validation dataset plus GEPIA). It is noteworthy that each gene whose expressional alteration has been confirmed in both COAD and READ cancer types by the GEPIA were considered 1) to have previously known or 2) to be most contingent for having participation in colorectal cancer. In the present study, we became prosperous to announce 13 vDEGs whose expressional participation in colorectal cancer has not been previously declared, including 1) over-expressed ones (as possible oncogenes in colorectal cancer): MFAP2, CELSR3, and 2) under-expressed ones (as possible tumor suppressor genes in colorectal cancer): P2RY14, NPY1R, ARL14, TNXB, IGLV1-44, UGT2B15, IGHA2, IL1R2, PCK1, SOSTDC1, and UGT2A3. Surprisingly, the specificity and sensitivity of reported novel genes, using the expression values of all 186 patients and 54 healthy samples in GSE41258, were sufficiently high in distinguishing healthy individuals from CRC patients (Fig 23). Regarding the detected novel genes, more details from other research works are presented in the following.

MFAP2 has the potential to be considered an oncogene, triggering EMT and causing cancer progression, with diagnostic capability in gastric cancer. Bearing the prognostic value in mind, MFAP2 serves as a negative indicator for overall survival in gastric cancer. Also, this gene is observed increased in head and neck squamous carcinoma, particularly in lymph node metastasis [30, 31]. MFAP2 gene shares its oncogenic role not only in gastric cancer [32] but also in breast cancer. Restriction of MFAP2 can culminate in inhibition of proliferation, migration, and invasion in BC cells [33]. MFAP2 has the ability to determine the phenotype of cancer cells through altering ECM in cancer microenvironment. By the same token, this gene can lead to unpleasant outcomes among patients with liver cancer, pancreatic cancer, renal cancer, and cervical cancer [34].

The expression of ARL14 corresponds positively to prognosis in bladder cancer [35].

The down-regulation of TNXB is observed in cervical cancer and its up-regulation is known as correlated with the inhibition of EMT, migration, and invasion in this cancer [36]. TNXB is down-regulated in endometrial cancer, as well [37].

UGT2B15, as an oncogene, plays a main art in cancer progression, lymph node metastasis, and resistance to therapeutics in prostate cancer. More specifically, this gene can decrease the chance of responding to tamoxifen [38] and castration in breast and prostate cancers, respectively. Additionally, it is previously reported that UGT2B15 is enhanced in gastric cancer [39]. By the same token, the increased level of UGT2B15 expression exists in some subtypes of breast cancer accompanying a better survival rate [38].

In the light of prognosis, IGHA2 is tied in with basal-like and triple-negative breast cancer [40].

It is already proposed that IL1R2 is potentiated to act as an oncogene in that the high level of its expression, through inflammation and angiogenesis, culminated in CRC progression and development. On the other hand, the reduced level of this gene is reported in atherosclerotic lesions [41, 42].

While the over-expression of PCK1 was declared in colon cancer [43], lung cancer [44], melanoma [45], and lymphoma [43], metastatic breast cancer cells [43, 45, 46], and skin cancer [47], the down-regulation of this gene is present in kidney cancer and hepatocellular carcinoma (HCC) [43, 47, 48]. To be more elaborated, PCK1 promotes apoptosis and restrains both proliferation and tumor growth in HCC [43]. On the other hand, the expression of PCK1 is sometimes considered a factor leading to increased proliferation [44, 47, 49, 50].

SOSTDC1, through its down-regulation, provokes poor prognosis, tumor invasion, and low survival rate in multiple cancer types [51–54]. Furthermore, the raised expression level of SOSTDC1 can inhibit tumor growth in gastric cancer [55]. In thyroid cancer, breast cancer, and non-small cell lung carcinoma (NSCLC), SOSTDC1 being able to restrict proliferation is under-expressed [52, 53, 56–60]. In NSCLC, while the down-regulated SOSTDC1 can produce poor prognosis, its up-regulation is capable of inhibiting proliferation, migration, and EMT [53].

A low survival rate was observed in colon cancer patients bearing a high expression level of UGT2A3 [61].

In the ground of methylation, one hyper-methylated/under-expressed gene (CA12) and two hypo-methylated/over-expressed genes (COL1A1, and TACSTD2) were found which were confirmed both by expression (only by the validation dataset) and methylation (by MEXPRESS in two COAD, and READ cancer types). However, we had a chance of detecting three noticeably hyper-methylated/under-expressed genes (TCF21, MYH1, and DES) and seven outstandingly hypo-methylated/over-expressed genes (CCL20, CEMIP, CLDN1, KRT23, MMP7, SLC6A6, and SLC7A5) which not only were among v-DEGs-DMGs but also, pursuant to the GEPIA and the MEXPRESS they are of verified expressional and methylational value in both COAD and READ cancer types. It is worth mentioning that any gene whose methylation change has been demonstrated in both COAD and READ by the MEXPRESS was considered 1) to have previously known or 2) to be most probable for having a role in colorectal cancer. CHP2, as a hyper-methylated/under-expressed gene, had a statistically significant inverse/negative relationship between its expression and methylation. While the expressional alteration of CHP2 gene has been validated by the validation dataset its methylation status was confirmed only in COAD cancer type by the MEXPRESS. Taking all afore-mentioned issues and the appropriate ROC curve of CHP2 into account, we propose the hyper-methylation of this gene as a novel probable diagnostic marker in colorectal cancer. Furthermore, MSLN, as a hypo-methylated/over-expressed gene, illustrated a statistically significant inverse/negative relationship between its expression and methylation levels. Considering firstly, the GEPIA-verified expression change of MSLN gene in both COAD and READ and secondly, not being MEXPRESS-verified methylation status of this gene and for third, its plausible ROC curve we introduce the hypo-methylation of this gene as a contingent diagnostic marker in colorectal cancer for the first time. In the continuation of this research study, among vDEGs-DMGs, we found five (CHP2, CXCL12, EPB41L3, SCNN1B, and PYY) and one (MUC4) hyper-methylated/under-expressed genes which were corroborated in COAD and READ by the MEXPRESS, respectively. These are noteworthy that CXCL12 and EPB41L3 were verified by the GEPIA in COAD and READ cancer types while SCNN1B and PYY were documented only in COAD cancer type by the GEPIA. MUC4 and CHP2 genes have been validated solely by the validation dataset. By the same token, among vDEGs-DMGs, we discovered four (CST1, CHI3L1, UBD, and ASCL2) and two (INHBA, and ITGBL1) hypo-methylated/over-expressed genes which were recognized in COAD and READ by the MEXPRESS, respectively. These are worthwhile to point out that CST1, CHI3L1, UBD, and INHBA genes were verified by the GEPIA in both COAD and READ cancer types while ASCL2 was verified only in COAD cancer type by the GEPIA. ITGBL1 gene has been validated just by the validation dataset.

Pursuant to the correlation plots in this study, there are several genes with an inverse/inverse correlation between their expression and methylation values which were not statistically significant. We conjecture that the reasons behind this fact include 1) the limited number of patients, and 2) the methylation is not the main regulatory system and instead, the alternative mechanism(s) is/are regulating the expression of these genes. By the same token, changing in methylation does not necessarily end up measurable expression alteration. Even in several studies, the positive or direct correlation between gene expression and methylation in the gene body has been reported [22].

In the comparison between the power of expression and methylation profiles, the ROC curves elucidated that while both profiles are adequately sensitive and specific for diagnosis, the expression profile outruns the methylation profile to some extent.

With respect to the genes having interaction(s) with miRNAs, we realized four down-regulated vDEGs (TMEM100, MYH11, CHRDL1, and FAM107A) to the accompaniment of five up-regulated vDEGs (KLK10, CLDN1, SLC6A6, MMP11, and SLC7A5) being documented by the GEPIA in both COAD and READ cancer types. Ultimately, we ascertained one down-regulated vDEG (MYH11) and three up-regulated vDEGs (SLC7A5, SLC6A6, and CLDN1) which are under the dual regulatory mechanisms; miRNAs, and methylation. The expression and the methylation status of the last-mentioned genes have been corroborated by the GEPIA (COAD and READ) and the MEXPRESS (COAD and READ).

## 4. Conclusion

In conclusion, we averred four and one findings pertaining to the diagnosis and prognosis of colorectal cancer, respectively. Taking the diagnosis into account, an expression profile containing 43 over-expressed vDEGs and 91 under-expressed vDEGs, two of which and 11 of which were novel in CRC respectively, was detected. It is worth re-announcing that two of 11 novel under-expressed vDEGs were included in the hub genes. By the same token, a methylation profile including 22 hypo-methylated/over-expressed vDEGs-DMGs and 15 hyper-methylated/under-expressed vDEGs-DMGs, in per of which one novel differentially methylated gene was discerned, was introduced. According to the ROC curves, in the comparison between the diagnosis power of the expression profile and that of the methylation profile the more specificity and sensitivity for the expression profile were established as for CRC. The recognized miRNA expression profile demonstrated six over-expressed vDEGs interacting with six under-expressed DEMs and nine down-regulated vDEGs interacting with eight up-regulated DEMs. Taking the issue of prognosis into consideration, eight over-expressed vDEGs to the accompaniment of 15 under-expressed vDEGs were ascertained playing a crucial role in determining the survival time as for CRC patients. An outstanding point is that the declared profiles, particularly their novel genes due to their high enough powers in diagnosis, are capable of serving in targeted therapy. However, our results need to be more confirmed by further research studies, particularly clinically, due to two obstructive circumstances which should be tackled; the confined number of patients, and solely-bioinformatics-based analysis.

## 5. Materials and methods

### 5.1. Searching for suitable microarray data

Searching in the Gene Expression Omnibus (GEO, http://www.ncbi.nlm.nih.gov/geo) database, and using “Colorectal Neoplasm”, “Case/Control” or “Primary/Normal”, “Homo Sapiens”, and “Expression Profiling by Array” or “Methylation Profiling by Array” as “MeSH Terms”, “All Fields”, “Organism”, and “Study Type”, respectively, we looked for appropriate array datasets. It is noteworthy that in this step, “Tumor Stage”, “Metastasis”, and “any types of intervention” were not considered the determining criteria. Ultimately, we found three array datasets including, 1) GSE41258 [62, 63] (GPL96 [HG-U133A] Affymetrix Human Genome U133A Array]) as a testing or discovery expression array dataset with tissue samples from 54 healthy participants and 186 colorectal cancer patients, 2) GSE101764 [64] (GPL13534 [Illumina HumanMethylation450 BeadChip (HumanMethylation450_15017482)]) as a testing methylation array dataset with tissue samples having obtained from 149 healthy participants and 112 colorectal cancer patients, and 3) GSE31905 [65] (GPL6480 [Agilent-014850 Whole Human Genome Microarray 4x44K G4112F (Probe Name version)]) as a validating expression array dataset with specimens from seven healthy and 55 colorectal cancer patients.

### 5.2. Identification of Differentially Expressed Genes (DEGs) and Differentially Methylated Genes (DMGs)

**Fig 24:**
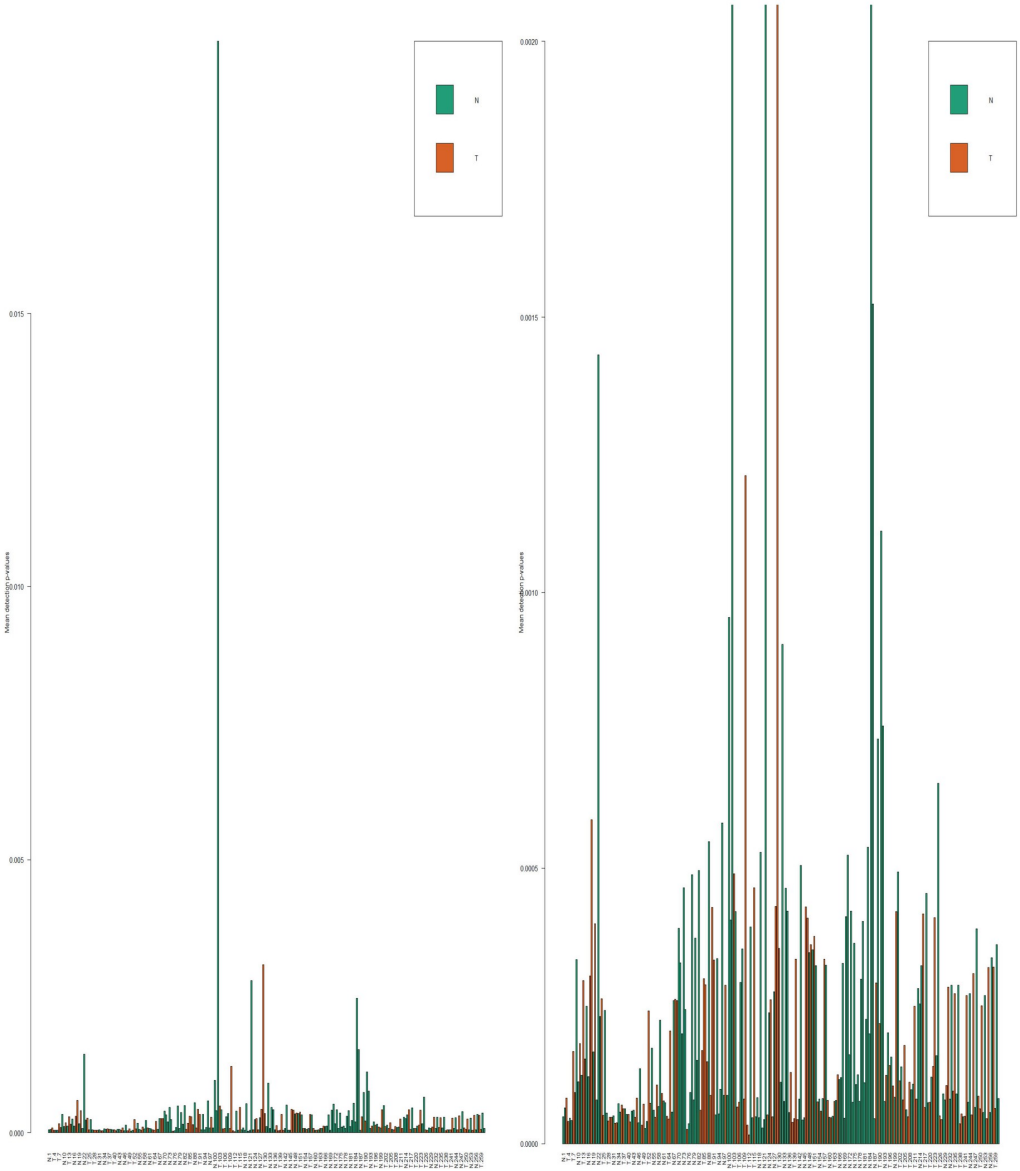
The detected P.values of all samples. The right side is the zoomed model of the left side. As it is shown all samples are in statistically significant range.

Utilizing GEO2R, an online differentially analyzing tool, and “GEOquery” (Version 2.54.0) [66], and “Limma” (Version 3.42.0) [67] R (Version 3.6.3) packages, we exerted log2 transformation and Benjamini & Hochberg method on GSE41258 to find the differentially expressed genes in a case/control comparison manner. By the same token, |LogFC| ≥ 2 and FDR-adj.P.value < 0.05 were the thresholds to define statistical significant results. In view of the satisfactory distribution of the samples’ box plot’s medians, the discovery expression dataset (GSE41258) did not need to be more normalized. Also, we took advantage of “Limma”, “minfi (Version 1.32.0)” [68–74], missMethyl (Version 1.20.4)” [69, 75–77], “minfiData (Version 0.32.0)”, “DMRcate (Version 2.0.0)” [78], “IlluminaHumanMethylation450kanno.ilmn12.hg19 (Version 0.6.0)”, and “IlluminaHumanMethylation450kmanifest (Version 0.4.0)” R packages in order to detect the differentially methylated genes out of GSE101764. So as to perform DMGs, the raw data (IDAT files) of the methylation array dataset was downloaded. We visualized samples according to their quality and then the samples whose P.values were less than 0.05 were preserved (Fig 24). Through the PreprocessFunnorm function, the normalization was exerted. The probes whose qualities were with P.value < 0.01 were kept in the study. Additionally, since our samples were taken from both sexualities we proceeded to omit the probes located on the sex chromosomes. In the next steps, we excluded the probes contained SNPs in CpG islands and the cross-reactive probes. Finally, an unpaired-analysis was conducted in a case/control comparison manner, and the probes with FDR < 0.05 being statistically significant were maintained in the present research study. Then, we converted the approved probes into the genes on which they are located, and by defining |LogFC| ≥ 1 and adj.P.value < 0.05 as thresholds the statistically significant results were introduced. It is noteworthy, we used false discovery rate adjusted P.values through our differential analyses to avoid type I error in multiple testing.

### 5.3. Validation of Differentially Expressed Genes

Consistent with analyzing strategy (|LogFC| ≥ 2 and FDR-adj.P.value < 0.05) in the testing or discovery expression array dataset, we validated the differentially expressed genes using GSE31905. It is noteworthy that through using the normalizeQuantiles function, the normalization process has been performed to preserve the comparability status. Through the Venny (Version 2.1.0) (https://bioinfogp.cnb.csic.es/tools/venny/) [79] and plotting Venn diagram, we intersected differentially expressed genes between testing and validating expression array datasets.

### 5.4. Identification of validated Differentially Expressed Genes (vDEGs)-Differentially Methylated Genes (DMGs)

Taking advantage of the Venny diagrams, we attempted to discover 1) over-expressed vDEGs-hypo-methylated DMGs and 2) under-expressed vDEGs-hyper-methylated DMGs. Furthermore, by plotting heatmap (ClustVis, https://biit.cs.ut.ee/clustvis/) [80] for the above-mentioned gene groups, we investigated the pattern through which the expressional levels of those genes are changed between normal and cancer groups. These are worth mentioning that firstly, the expression values used in heatmap plots were obtained from the testing expression array dataset (GSE41258), and secondly, the heatmap plots were drawn pursuant to correlation. By the same token, we drew another heatmap plot based on the TCGA data using the UALCAN database (http://ualcan.path.uab.edu/index.html) [81].

### 5.5. Verification of validated Differentially Expressed Genes (DEGs)-Differentially Methylated Genes (DMGs) by MEXPRESS and TCGA data

MEXPRESS (https://mexpress.be/) is an online tool for visualizing expression and methylation using TCGA data. Using the MEXPRESS tool, we tried to validate the vDEGs-DMGs based on TCGA data. The criteria we observed in the MEXPRESS were including 1) Omitting the samples whose methylation levels were characterized as null, 2) ordering the samples according to their expressional levels, 3) existence or non-existence of neoadjuvant therapy (as the sole clinical parameter), and 4) performing analysis on colon (COAD) and rectum (READ) cancer types data in TCGA database. So as to verify the DMGs through the MEXPRESS, we considered the existence of a statistically significant inverse/negative relationship between expression and methylation levels of most of the probes in each individual gene.

### 5.6. Expression-methylation correlation plots for validated Differentially Expressed Genes (vDEGs)-Differentially Methylated Genes (DMGs)

With the produced expression and methylation values obtained from GSE41258 and GSE101764 datasets, respectively, the correlation plots were drawn for normal and cancer participants, separately in order to perform the comparisons more precisely. Since the numbers of patients and normal samples in the above-mentioned datasets are not identical, the redundant patients and healthy samples were excluded randomly out of GSE41258 and GSE101764, respectively in order to match. The negative correlations with P.value < 0.05 were considered statistically significant.

### 5.7. Receiver operating characteristic curves for validated Differentially Expressed Genes (vDEGs)-Differentially Methylated Genes (DMGs)

Via the easyROC (Version 1.3.1) (http://www.biosoft.hacettepe.edu.tr/easyROC/), a web tool for ROC curve analysis, and using the expression and methylation values from GSE41258 and GSE101764 datasets, respectively we drew ROC curves for vDEGs-DMGs and for genes whose expression-methylation correlations were inverse/negative in the patients’ population. ROC curves were plotted as well for the efficacy of methylation and expression in this disease, separately. Accordingly, higher values in the differentially hyper-methylated and the differentially over-expressed genes were considered the indicators of higher risk. On the other hand, as for the differentially hypo-methylated and the differentially under-expressed genes, the lower values were assumed as the higher risk predisposing hallmarks. Analogous to the correlation plot, the number of patients and normal samples included in ROC curve analysis were set 112 and 54, respectively. The ultimate interpretation of the statistically significant results was based on the area under the ROC curve (AUC) and P.value < 0.05.

### 5.8. Identification of Differentially Expressed MicroRNAs (DEMs)

The dbDEMC 2.0 database (https://www.picb.ac.cn/dbDEMC/) [82], provides information regarding differentially expressed microRNAs obtained from the newly-released GEO and TCGA datasets in various cancer types. We utilized dbDEMC 2.0 in order to find DEMs in colorectal cancer patients in comparison with normal participants. Thereby, only datasets which included the comparisons between the studied groups were selected (GSE10289 [59 cases, and 7 controls], GSE30454 [54 cases, and 20 controls], GSE35602 [17 cases, and 4 controls], GSE35982 [8 cases, and 8 controls], GSE41012 [20 cases, and 15 controls], and GSE54088 [9 cases, and 10 controls] with 167 patients and 64 normal samples overall). Then, the DEMs with |LogFC| ≥ 2 were opted for remaining analyses.

### 5.9. Validated Differentially Expressed Genes (vDEGs)-Differentially Expressed MicroRNAs (DEMs) interactions

The miRTarBase database (http://mirtarbase.cuhk.edu.cn/php/index.php) [83], facilitating our way to detect interactions among genes and microRNAs, assisted us in finding the interactions connecting 1) the over-expressed vDEGs to under-expressed DEMs, and 2) the under-expressed vDEGs to over-expressed DEMs. Via the Cytoscape software (Version 3.7.2) those interactions became visualized.

### 5.10. More investigation of validated Differentially Expressed Genes (vDEGs) by GEPIA and TCGA data

The Gene Expression Profiling Interactive Analysis (GEPIA) database (http://gepia.cancer-pku.cn/) [84], paved the way for us to scrutinize the detected vDEGs in TCGA database. COAD and READ cancer types in TCGA database comprised our patient group and our normal population consisted of TCGA normal to the accompaniment of GTEx data. Following the LogScale transformation, |LogFC| ≥ 2 and P.value < 0.05 were determined as the statistically significant thresholds.

### 5.11. PPI networks construction and hub genes

The validated differentially expressed genes were investigated by the STRING database (Version 11.0) (https://string-db.org/) [85] to recognize the protein-protein interactions [4] among them. The median confidence of 0.4 was set and the nodes with no connections were ignored. Then, through using the Cytohubba plugin in the Cytoscape software and considering some pivotal benchmarks including, 1) co-expression, 2) homology, 3) experimentally determined interaction, 4) database annotated, and 5) combined score, the top ten hub genes were revealed and visualized.

### 5.12. Functional enrichment analysis of validated Differentially Expressed Genes (vDEGs)

The box plots and cnet plots for biological process (BP), molecular function (MF), and cellular component [86] were drawn regarding the vDEGs. The afore-mentioned Gene Ontology (GO) analyses were conducted and visualized by DOSE (Version 3.12.0) [87], enrichplot (Version 1.6.1), and clusterProfiler (Version 3.14.3) [88] packages in R software. Taking advantage of the Database for Annotation, Visualization and Integrated Discovery (DAVID) (Version 6.7) (https://david.ncifcrf.gov/), we discerned the Kyoto Encyclopedia of Genes and Genomes (KEGG) pathways with P.value < 0.05 the visualization of which was performed using Microsoft Excel 2016.

### 5.13. Survival analysis

The GEPIA database has also the ability to demonstrate survival plots for genes in various cancer types based on TCGA database. Accordingly, we combined COAD and READ patients groups to unravel the prognostic values of the detected vDEGs. The main characteristics of the survival plots are 1) using the Hazard Ratio (HR) with 95% Confidence Interval (CI), 2) considering the overall survival for patients, and 3) setting “Median” as the group cut off.

## Supporting information

S1 Table Over-expressed and under-expressed Differentially Expressed Genes obtained from the discovery dataset (GSE41258). (XLSX)

S2 Table Hypo-methylated and hyper-methylated Differentially Methylated Genes obtained from (GSE101764). (XLSX)

S3 Table Over-expressed and under-expressed Differentially Expressed Genes obtained from the validation dataset (GSE31905). (XLSX)

S4 Table Down-regulated Differentially Expressed MiRNAs obtained from the dbDEMC 2.0 database. (XLSX)
